# Lymphoma cells lacking pro-apoptotic BAX are highly resistant to BH3-mimetics targeting pro-survival MCL-1 but retain sensitivity to conventional DNA-damaging drugs

**DOI:** 10.1038/s41418-023-01117-0

**Published:** 2023-02-08

**Authors:** Sarah T. Diepstraten, Savannah Young, John E. La Marca, Zilu Wang, Ruth M. Kluck, Andreas Strasser, Gemma L. Kelly

**Affiliations:** 1 The Walter and Eliza Hall Institute of Medical Research, Melbourne, VIC Australia; 2 Department of Medical Biology, University of Melbourne, Melbourne, VIC Australia

**Keywords:** Cancer models, Preclinical research

## Abstract

BH3-mimetic drugs are an anti-cancer therapy that can induce apoptosis in malignant cells by directly binding and inhibiting pro-survival proteins of the BCL-2 family. The BH3-mimetic drug venetoclax, which targets BCL-2, has been approved for the treatment of chronic lymphocytic leukaemia and acute myeloid leukaemia by regulatory authorities worldwide. However, while most patients initially respond well, resistance and relapse while on this drug is an emerging and critical issue in the clinic. Though some studies have begun uncovering the factors involved in resistance to BCL-2-targeting BH3-mimetic drugs, little focus has been applied to pre-emptively tackle resistance for the next generation of BH3-mimetic drugs targeting MCL-1, which are now in clinical trials for diverse blood cancers. Therefore, using pre-clinical mouse and human models of aggressive lymphoma, we sought to predict factors likely to contribute to the development of resistance in patients receiving MCL-1-targeting BH3-mimetic drugs. First, we performed multiple whole genome CRISPR/Cas9 KO screens and identified that loss of the pro-apoptotic effector protein BAX, but not its close relative BAK, could confer resistance to MCL-1-targeting BH3-mimetic drugs in both short-term and long-term treatment regimens, even in lymphoma cells lacking the tumour suppressor TRP53. Furthermore, we found that mouse *Eµ-Myc* lymphoma cells selected for loss of BAX, as well as upregulation of the untargeted pro-survival BCL-2 family proteins BCL-XL and A1, when made naturally resistant to MCL-1 inhibitors by culturing them in increasing doses of drug over time, a situation mimicking the clinical application of these drugs. Finally, we identified therapeutic approaches which could overcome these two methods of resistance: the use of chemotherapeutic drugs or combined BH3-mimetic treatment, respectively. Collectively, these results uncover some key factors likely to cause resistance to MCL-1 inhibition in the clinic and suggest rational therapeutic strategies to overcome resistance that should be investigated further.

## Introduction

The BCL-2 family-regulated (a.k.a. intrinsic, mitochondrial) pathway to apoptosis is critical for the removal of damaged or unwanted cells, and the killing of cells in response to many anti-cancer agents [1, 2]. Following a death stimulus, the levels of BH3-only proteins (e.g., BIM, NOXA, PUMA, BMF) are increased and these critical inducers of apoptosis bind via their BH3 domain into the hydrophobic pocket of the pro-survival BCL-2 family proteins (BCL-2, MCL-1, BCL-XL, BCL-W, A1/BFL-1). Some BH3-only proteins (e.g., PUMA, BIM) have also been reported to activate the pro-apoptotic effector proteins BAX and BAK directly [1, 2]. Together these events allow activated BAX and BAK to oligomerise and form pores in the mitochondrial outer membrane. Release of apoptogenic factors from the mitochondria leads to the activation of caspases and, ultimately, dismantling of the cell [3].

Direct activation of the intrinsic apoptotic pathway using drugs termed BH3-mimetics has shown success for therapy of certain blood cancers [4, 5]. These drugs mimic the interaction of the BH3 domain from the pro-apoptotic BH3-only proteins with the hydrophobic groove of the pro-survival BCL-2 proteins, thereby allowing activated BAX and BAK to induce apoptosis [5, 6]. The most clinically advanced BH3-mimetic drug is the BCL-2-specific inhibitor venetoclax, which is approved by the FDA and many other international regulatory authorities for therapy of chronic lymphocytic leukemia (CLL) and acute myeloid leukemia (AML) [7–9]. BH3-mimetic drugs targeting related pro-survival BCL-2 proteins have also been developed for cancer therapy. A wealth of evidence supports the notion that MCL-1 inhibitors would be efficacious for a broad range of haematological malignancies, including for aggressive MYC-driven lymphomas, such as Burkitt Lymphomas (BL), which are known to be highly dependent on MCL-1 for continued survival and proliferation [10–16]. Such MYC-driven lymphomas can be modelled pre-clinically using the *Eµ-Myc* transgenic mouse model, which recapitulates the *immunoglobulin/c-MYC* chromosomal translocation characteristic of BL, and results in pre-B/B cell lymphomas with almost 100% incidence [17]. When these mouse lymphoma cells are transplanted into recipient mice, loss of even one allele of *Mcl-1* can invoke tumour regression, demonstrating their high dependence on MCL-1 for sustained survival and tumour expansion [14]. There is also evidence that MCL-1-targeting BH3-mimetics could be effective for certain solid cancers when used alongside inhibitors of oncogenic kinases [18–20]. The clinical progression of MCL-1-targeting BH3-mimetics has been limited by on-target toxicity, since MCL-1 is required for the survival of many healthy cell types, including cardiomyocytes [5]. Nevertheless, evidence suggests a therapeutic window for MCL-1 inhibitors can be achieved, where tumour cells are killed but healthy cells are spared [20]. As such, six MCL-1 inhibitors have entered into stage 1 clinical trials for relapsed/refractory lymphomas, AML and multiple myeloma [5].

Recent evidence from clinical trials with venetoclax in CLL and AML patients has shown that drug resistance can emerge over time despite initial complete responses [21–23]. Therefore, understanding the underlying mechanisms of resistance to BH3-mimetic drugs is critical to improve therapeutic outcomes in patients by developing rational drug combination therapies. In previous studies using cell lines and patient samples derived from diverse blood cancers, we identified loss of the tumour suppressor TP53 as a resistance factor to single agent therapy using either BCL-2-specific or MCL-1-specific BH3-mimetic drugs [24]. This was consistent with the analysis of CLL and AML patients that relapsed on venetoclax therapy, as well as with other published studies seeking to identify resistance factors to BCL-2 inhibitors [22, 23, 25–29]. Whilst performing CRISPR/Cas9 whole genome knockout (KO) screens to identify factors that confer resistance to MCL-1-targeting BH3-mimetics in *Eµ-Myc* lymphoma cells, we additionally identified loss of the apoptotic effector BAX as a top hit [24]. Here we delve deeper into this result, showing that loss of BAX is also a hit when such screens are performed in *Trp53*-deficient *Eµ-Myc* lymphoma cells, and additionally arises as the key mediator of resistance in longer-term CRISPR/Cas9 gene KO screens using suboptimal doses of MCL-1 inhibitors. Loss of BAX expression is also a prominent feature in *Eµ-Myc* lymphoma cells selected to be BH3-mimetic drug resistant through culturing for extended periods in increasing doses of these agents. These data reveal that BAX, but not BAK, is the key mediator of optimal MCL-1 inhibitor-induced killing of malignant cells. Importantly, such BAX-deficient lymphoma cells, unlike TRP53-deficient lymphoma cells, can still be potently killed by standard chemotherapeutic drugs, indicating a potential therapeutic strategy to overcome this mode of resistance to BH3-mimetic drugs.

## Results

### Whole genome CRISPR/Cas9 gene knockout screens consistently identify loss of BAX as a resistance factor to MCL-1-targeting BH3-mimetic drugs in *Eµ-Myc* mouse lymphoma cells

**Fig. 1:**
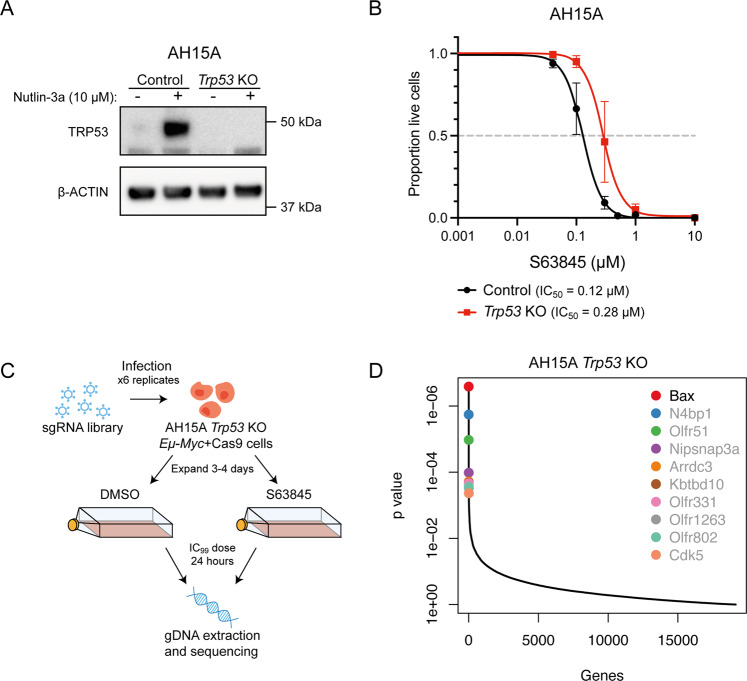
Whole genome CRISPR/Cas9 KO screens reveal that loss of BAX confers resistance to BH3-mimetic drugs targeting MCL-1 in *Eµ-Myc* mouse lymphoma cell lines with *Trp53* KO. **A** Western blot to validate CRISPR/Cas9-mediated KO of *Trp53* in AH15A *Eµ-Myc* mouse lymphoma cells. Cells were treated with the TRP53-activating drug Nutlin-3a for 24 h to enable visualisation of stabilised wildtype TRP53 protein in *Trp53* wildtype cells. Probing for β-ACTIN served as a loading control. **B** Dose–response curves for control AH15A *Eµ-Myc* lymphoma cells (containing Cas9 and a non-targeting control sgRNA) and isogenic *Trp53* KO cells treated with the MCL-1 inhibitor S63845 for 24 h. Live cells were identified as Annexin V/PI double negative by flow cytometry. *Trp53* KO cells showed a ~2-fold increase in IC_50_ for S63845 treatment. **C** Schematic of CRISPR/Cas9 screen performed in the AH15A *Trp53* KO mouse *Eµ-Myc* lymphoma cell line. Cells were treated with DMSO (vehicle, control) or 1 µM of S63845 (~IC_99_ dose) for 24 h and surviving cells were collected for next-generation sequencing. **D** MAGeCK analysis to identify top enriched sgRNAs in S63845-resistant cell populations. Significant hits (FDR < 0.05) are indicated with black text. Loss of *Bax* was identified as the top, and only significant, hit that promoted drug resistance in the AH15A *Trp53* KO *Eµ-Myc* lymphoma cell line.

We previously identified loss of TRP53 as a factor which could confer resistance to MCL-1-targeting BH3-mimetic drugs in short-term CRISPR/Cas9 KO screens performed in *Eµ-Myc* mouse lymphoma cells [24]. To explore this further, we generated a *Trp53* KO *Eµ-Myc* lymphoma cell line (AH15A *Trp53* KO) using CRISPR/Cas9 gene editing, and validated loss of TRP53 by western blotting (Fig. 1A). Despite being the top hit identified in these previous screens, we found that loss of TRP53 provided only modest resistance to MCL-1 inhibitors during short-term killing assays (24 h) (Fig. 1B). Therefore, as many human cancers have TP53 mutations, we sought to identify additional factors that might enable TRP53-deficient lymphoma cells to resist high doses of BH3-mimetics. To this end, we performed a whole genome CRISPR/Cas9 KO screen in *Trp53* KO cells using the mouse YUSA sgRNA library [30] (Fig. 1C). We treated library-infected, Cas9-expressing cells with a high dose of the MCL-1-targeting BH3-mimetic S63845 (IC_99_) for a short timeframe (24 h) and found that sgRNAs targeting the pro-apoptotic BCL-2 family gene *Bax* were significantly enriched by drug treatment (Fig. 1D). No other significant hits were identified from this screen, suggesting that loss of TRP53 and BAX are the most potent resistance factors for MCL-1 inhibitors.

**Fig. 2:**
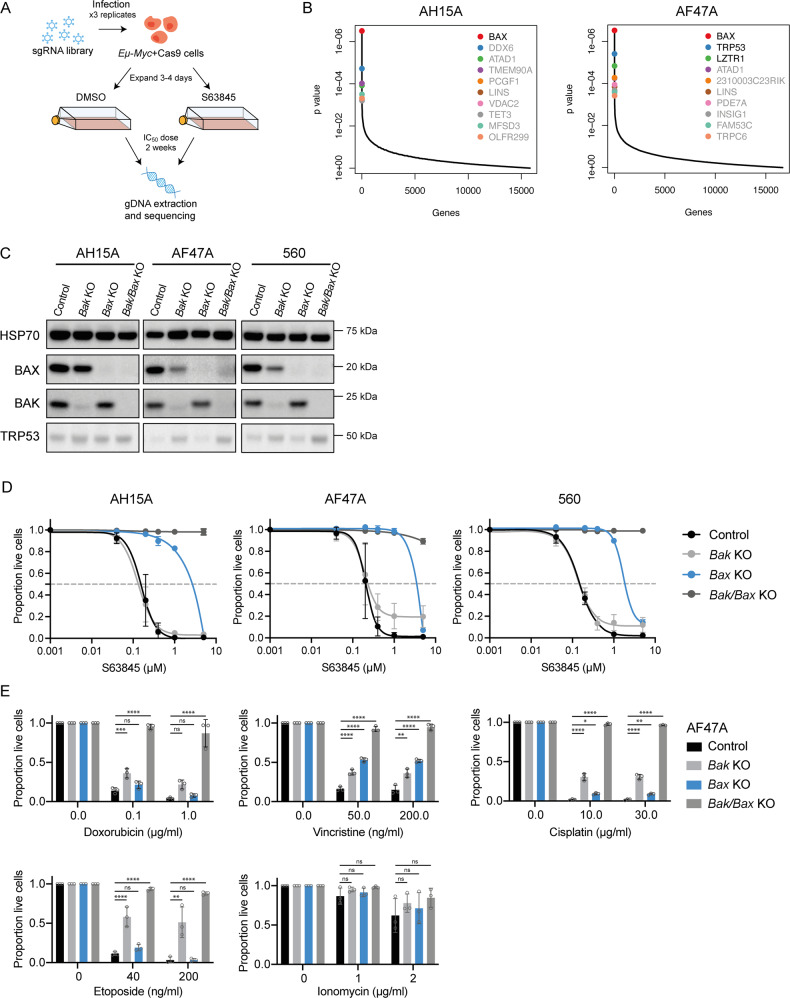
Whole genome CRISPR/Cas9 KO screens reveal loss of *Bax* as the top factor conferring resistance to BH3-mimetic drugs targeting MCL-1 in *Eµ-Myc* mouse lymphoma cell lines. **A** Schematic of CRISPR/Cas9 screen performed in *Eµ-Myc* lymphoma cells with wildtype *Trp53*. Cells were treated with DMSO (vehicle, control) or 100 nM of S63845 (~IC_50_ dose) for 2 weeks and surviving cells were collected for next-generation sequencing. **B** MAGeCK analysis to identify top enriched sgRNAs in S63845-resistant cell populations. Significant hits (FDR < 0.05) are indicated with black text. Loss of *Bax* was identified as the top hit in the two independent *Eµ-Myc* lymphoma cell lines used. **C** Western blots to validate CRISPR/Cas9-mediated loss of BAK, BAX or both BAK and BAX in three independent *Eµ-Myc* lymphoma cell lines. Probing for HSP70 served as a loading control. **D** Dose–response curves for control *Eµ-Myc* lymphoma cells (containing Cas9 and a non-targeting control sgRNA) and isogenic *Bak* as well as *Bax* single KO or *Bak*/*Bax* double KO cells treated with the MCL-1 inhibitor S63845 for 24 h. Live cells were identified as Annexin V/PI double negative by flow cytometry. **E** Isogenic WT, *Bak* KO, *Bax* KO or *Bak*/*Bax* double KO AF47A lymphoma cells treated with the cytotoxic drugs doxorubicin, vincristine, cisplatin, etoposide or ionomycin. All data are presented as mean ± SD for 3 independent experiments. One-way ANOVA was used to measure statistical significance (*p < 0.05, **p < 0.01, ***p < 0.001, ****p < 0.0001, ns = not significant).

We next aimed to identify factors that conferred resistance to suboptimal doses of S63845 over a longer timeframe, as this may be more representative of drug resistance that arises in patients (Fig. 2A). To this end, two independent, YUSA library-infected, Cas9-expressing *Eµ-Myc* lymphoma cell lines with wildtype *Trp53* expression were treated with suboptimal doses (<IC_50_) of S63845 for 2 weeks. Significant enrichment of sgRNAs targeting *Bax* were identified (Fig. 2B). As in the previous screen performed in *Trp53* KO cells, sgRNAs targeting the related pro-apoptotic gene *Bak* were not enriched after drug treatment. This indicates that loss of BAX, but not loss of BAK, confers resistance to MCL-1 inhibitors in these cells.

To confirm this result, we used CRISPR/Cas9 to generate isogenic *Eµ-Myc* lymphoma cell lines that lack either BAK, BAX, or both of these effectors of apoptosis, as confirmed by western blotting (Fig. 2C). When treated with S63845, *Bax* KO lymphoma cells showed a 10-fold increase in IC_50_ compared to cells containing non-targeting (control) sgRNAs (control cells; Fig. 2D). In contrast, loss of BAK did not confer protection from S63845. Interestingly, loss of both BAK and BAX profoundly protected cells from even high doses of S63845, beyond that of BAX loss alone. This reveals that whilst BAX is the primary mediator of the apoptotic response in *Eµ-Myc* lymphoma cells following exposure to MCL-1-targeting BH3-mimetic drugs, BAK does play an important complementary role in this apoptotic process.

### *Eµ-Myc* lymphoma cells lacking BAX expression can still be killed by cytotoxic drugs

One plausible explanation for our finding that loss of BAX but not loss of BAK confers resistance to MCL-1 inhibitors is that *Eµ-Myc* lymphoma cells depend only on BAX to undergo cell death. To determine whether loss of BAX conferred resistance selectively to S63845, or more generally to all cytotoxic drugs that induce apoptosis, isogenic *Bak* KO, *Bax* KO and *Bak/Bax* double KO cell lines from three independent *Eµ-Myc* lymphomas were generated using CRISPR/Cas9 gene editing and treated with standard-of-care agents that are part of the R-CHOP regimen, namely doxorubicin and vincristine [31]. In addition, we tested the sensitivity to cisplatin, which is used as salvage therapy for lymphoma patients [32] and also to the DNA-damaging agent etoposide as well as the calcium ionophore ionomycin (Fig. 2E and Supplementary Fig. 1). In all cases, loss of BAX conferred no substantial protection against these cytotoxic drugs compared to control cells, showing that loss of BAX is not a general resistance factor in these *Eµ-Myc* lymphoma cells, but confers specific resistance to S63845. In fact, the *Bak* KO *Eµ-Myc* lymphoma cells seemed more resistant to some of the standard-of-care drugs than the *Bax* KO lymphoma cells. The *Bak/Bax* double KO *Eµ-Myc* lymphoma cells were markedly resistant to all agents (Fig. 2E and Supplementary Fig. 1), confirming that these drugs kill these cells by inducing apoptosis.

**Fig. 3:**
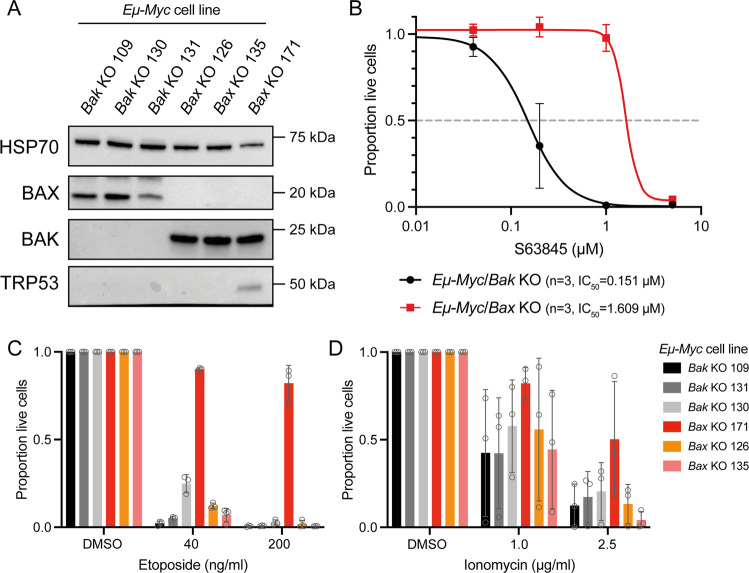
Lymphomas arising from *Eµ-Myc*/*B*ax KO mice show resistance to MCL-1-targeting BH3-mimetics but retain sensitivity to etoposide. **A** Cell lines were derived from lymphomas arising in *Eµ-Myc*/*Bak* KO and *Eµ-Myc*/*Bax* KO mice. Western blotting was used to validate the loss of BAK or BAX protein in these cell lines, respectively. Probing for HSP70 was used as a loading control. **B** Dose–response curves for *Eµ-Myc*/*Bak* KO and *Eµ-Myc*/*Bax* KO lymphoma cell lines treated with the MCL-1 inhibitor S63845 for 24 h. Live cells were identified as Annexin V/PI double negative by flow cytometry. Three independent cell lines were tested for each genotype. **C**, **D** Survival of *Eµ-Myc*/*Bak* KO and *Eµ-Myc*/*Bax* KO lymphoma cell lines treated with the cytotoxic drugs etoposide (**C**) or ionomycin (**D**). Loss of BAX provided no protection from these drugs. One *Eµ-Myc*/*Bax* KO cell line (171) showed resistance to etoposide treatment, likely due to the presence of an additional mutation in *Trp53*. All data are presented as mean ± SD for 3 independent experiments.

We confirmed these results by crossing *Eµ-Myc* transgenic mice to mice that had been genetically engineered to have *Bax* or *Bak* gene KO. Cell lines were established from independent lymphomas that arose in three sick *Eµ-Myc*/*Bak* KO and three sick *Eµ-Myc*/*Bax* KO mice (Fig. 3A). Western blotting for TRP53 in these cell lines revealed that one *Eµ-Myc*/*Bax* KO cell line (171) likely possessed a *Trp53* mutation, as indicated by stabilisation of TRP53 protein (Fig. 3A). Similar to the results in the CRISPR/Cas9-edited cells, *Eµ-Myc*/*Bax* KO cells were >10-fold more resistant to S63845 than *Eµ-Myc*/*Bak* KO cells (Fig. 3B). Neither lack of BAK nor lack of BAX protected these cells from etoposide or ionomycin (Fig. 3C, D). Together, these results confirm that conventional chemotherapies are still effective at killing BAX KO lymphoma cells that are resistant to S63845.

### Generation of MCL-1 inhibitor-resistant cells in culture selects for loss of BAX protein expression

**Fig. 4:**
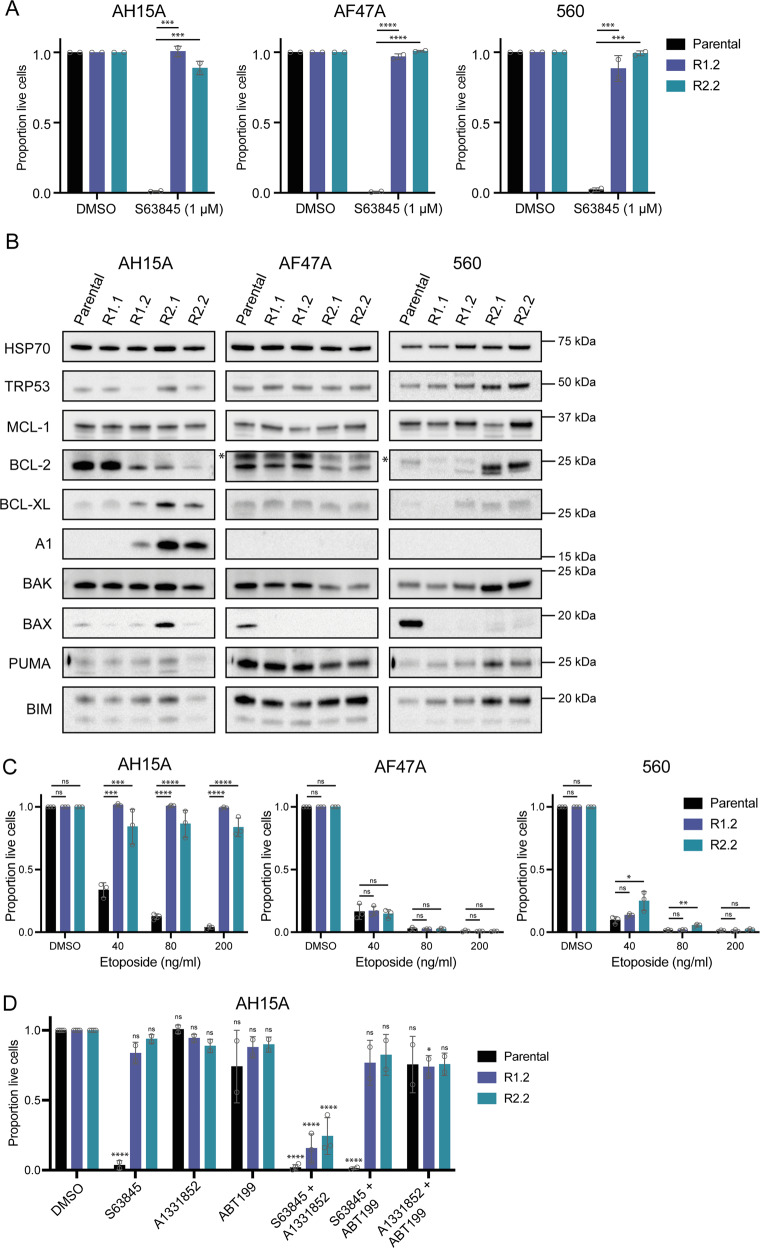
Generation and characterisation of MCL-1 inhibitor-resistant *Eµ-Myc* lymphoma cell lines. To generate MCL-1 inhibitor-resistant cells, three independent *Eµ-Myc* lymphoma cell lines were cultured in increasing amounts of S63845 over time, until they were resistant to 1 µM S63845 (10× IC_50_). Cell lines were then either collected for analysis (resistant cell lines R1.1 and R2.1) or continued to be cultured until they were resistant to 5 µM S63845 (resistant cell lines R1.2 and R2.2). Two independent culturing campaigns were undertaken. **A** Cell viability assay following treatment of parental and drug-resistant cell lines with 1 µM S63845 for 24 h. Live cells were identified as Annexin V/PI double negative by flow cytometry. **B** Western blotting for TRP53 as well as for pro-apoptotic and pro-survival BCL-2 family proteins in parental and drug-resistant cell lines. Probing for HSP70 was used as a loading control. * indicates non-specific bands. **C** Parental and drug-resistant cell lines derived from three independent *Eµ-Myc* lymphoma cell lines were treated with the chemotherapeutic drug etoposide for 24 h. Live cells were identified as Annexin V/PI double negative by flow cytometry. **D** Treatment of *Eµ-Myc* lymphoma cell lines exhibiting upregulated pro-survival proteins BCL-XL or A1 (AH15A R1.2, R2.2) with the BH3-mimetic drugs S63845 (MCL-1 inhibitor), A-1331852 (BCL-XL inhibitor) and venetoclax/ABT-199 (BCL-2 inhibitor), either alone or in the indicated combinations for 24 h. Live cells were identified as Annexin V/PI double negative by flow cytometry. In (**D**), significance is measured versus the DMSO-treated sample for each cell line. All data are presented as mean ± SD for 2–3 independent experiments. One-way ANOVA was used to measure statistical significance (*p < 0.05, **p < 0.01, ***p < 0.001, ****p < 0.0001, ns = not significant).

The second method we employed to identify factors that confer resistance to MCL-1 inhibitors was through the generation of naturally resistant cell populations. We cultured *Eµ-Myc* lymphoma cell lines in increasing doses of S63845 over time, until the cells were resistant to doses of >1 µM (10× the IC_50_ of S63845 in parental cells; Fig. 4A). Two independent culturing experiments were undertaken, resulting in a total of four drug-resistant derivatives for each independent *Eµ-Myc* cell line.

To explore the underlying molecular changes that were conferring resistance to S63845 in these cells, western blotting was performed for TRP53, as well as both pro- and anti-apoptotic BCL-2 family proteins (Fig. 4B). In resistant variants derived from two of the three *Eµ-Myc* lymphoma cell lines (AF47A and 560), the most common abnormality identified was loss of BAX protein expression. Resistant cells derived from one cell line (AH15A) did not exhibit loss of BAX, and in fact one of the derived S63845-resistant variants (R2.1) actually showed increased BAX protein expression compared to the parental cell line. Instead, the AH15A drug-resistant variants showed increased expression of the pro-survival BCL-2 family members BCL-XL and A1 that are not targeted by S63845. We found that the AF47A and 560-derived S63845-resistant cell lines that had lost expression of BAX protein could still be killed by the TRP53-activating drug Nutlin-3a, indicating that they retained TRP53 function (Supplementary Fig. 2). Importantly, these resistant cell lines also retained sensitivity to the chemotherapeutic drug etoposide (Fig. 4C), similar to the *Bax* KO *Eµ-Myc* lymphoma cells we examined (Figs. 2 and 3). In contrast, etoposide could not effectively kill the AH15A-derived S63845-resistant cell lines that had increased expression of BCL-XL and A1. However, these lymphoma cells could be killed by combined targeting of MCL-1 and BCL-XL using S63845 and the BCL-XL-specific BH3-mimetic drug A-1331852 (Fig. 4D). These results suggest that BCL-XL is the primary mediator of resistance in these cells over A1, but the lack of an A1-targeting BH3-mimetic precludes a firm conclusion in this regard. The data do indicate that co-targeting of compensatory pro-survival proteins, which are upregulated in response to prolonged BH3-mimetic drug treatment, could be an effective therapeutic strategy.

### Splicing mutations in the *Bax* gene were identified in some MCL-1 inhibitor-resistant *Eµ-Myc* lymphoma cells

**Fig. 5:**
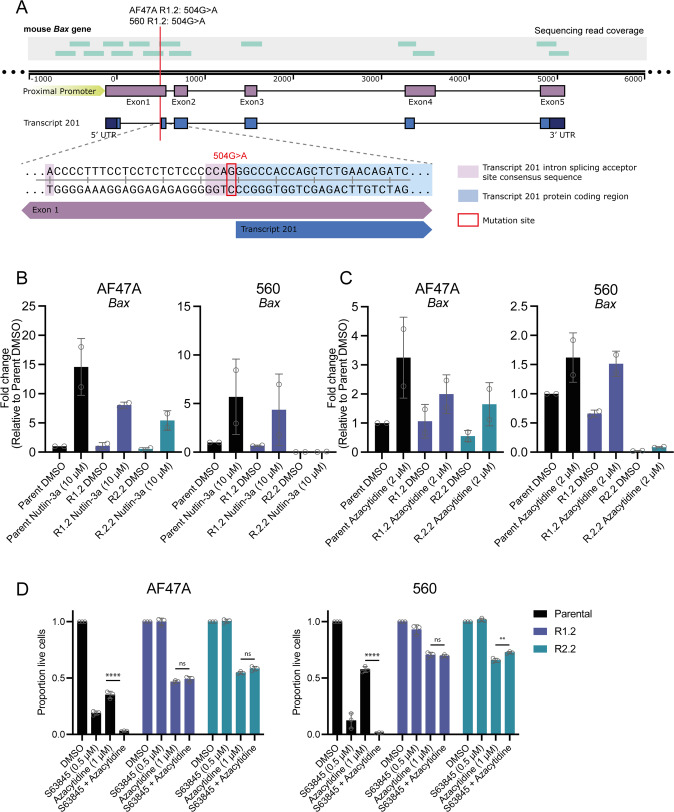
Various defects suppressing *Bax* expression were identified in MCL-1 inhibitor-resistant *Eµ-Myc* lymphoma cells. **A** Next-generation sequencing of the *Bax* coding regions revealed a 504G>A substitution downstream of the transcriptional start site in two drug-resistant *Eµ-Myc* lymphoma cell lines which lack BAX protein expression (AF47A R1.2 and 560 R1.2). This mutation disrupts the mRNA splicing acceptor site and would likely result in the retention of intron 1 in the consensus mouse *Bax* transcript *Bax-201* (ENSEMBL ENSMUST00000033093.10). Gene sequencing coverage is indicated by green bars. **B** qRT-PCR of parental and drug-resistant *Eµ-Myc* lymphoma cells treated with DMSO (vehicle, negative control) or the TRP53-activating drug Nutlin-3a for 24 h, of which *Bax* is a transcriptional target. Data are shown normalised to the housekeeping gene *Hmbs*, and relative to the parental *Eµ-Myc* lymphoma cell line treated with DMSO. **C** qRT-PCR of parental and drug-resistant *Eµ-Myc* lymphoma cells treated with DMSO or the hypomethylating agent 5’azacytidine (inhibitor of DNMT1) for 24 h. Data are shown normalised to the housekeeping gene *Hmbs*, and relative to the parental cell line treated with DMSO. **D** Parental and drug-resistant *Eµ-Myc* lymphoma cell lines exhibiting loss of BAX protein expression were treated with 5’azacytidine for 24 h, alone or in combination with S63845. Live cells were identified as Annexin V/PI double negative by flow cytometry. All flow cytometry data are presented as mean ± SD for 3 independent experiments. One-way ANOVA was used to determine statistical significance (**p < 0.01; ****p < 0.0001, ns = not significant). All qRT-PCR data are presented as mean ± SD for 2 independent experiments.

To investigate the underlying mechanisms that may have caused the loss of BAX protein expression in the S63845-resistant cells, next-generation sequencing of the *Bax* exons and proximal promoter was undertaken. This region of the *Bax* promoter contains important transcription factor binding sites, including for the tumour suppressor protein TRP53 [33]. Two of the four cell lines (AF47A R1.2 and 560 R1.2) examined were found to possess a G>A mutation of base pair 504 downstream of the transcriptional start site (Fig. 5A). This mutation disrupts the final base of the first mRNA intron consensus sequence splicing site of the canonical *Bax* transcript, *Bax-201*, which would likely result in this intron being retained in the mRNA. As we did not detect an elongated BAX protein in these cells by western blotting (Fig. 4B), it is likely that the mRNA encoding this mutant BAX protein is degraded, resulting in a BAX KO phenotype.

For the other resistant *Eµ-Myc* lymphoma cell lines that had lost BAX expression, we examined whether BAX may be dysregulated at the pre-transcriptional or post-transcriptional levels by performing quantitative reverse-transcriptase PCR (qRT-PCR) for *Bax* mRNA following treatment of the cells with the TRP53-activating drug Nutlin-3a (Fig. 5B). *Bax* is a known target gene of TRP53 and therefore, as expected, we observed an increase in *Bax* transcript levels in the parental *Eµ-Myc* lymphoma cell lines treated with Nutlin-3a compared to those treated with DMSO (vehicle, negative control). In the S63845 drug-resistant cell lines with *Bax* splicing mutations (AF47A R1.2 and 560 R1.2), as well as in AF47A R2.2, we saw upregulation of *Bax* mRNA following treatment with Nutlin-3a. However, in the 560 R2.2 lymphoma cells, no induction of *Bax* mRNA was observed after Nutlin-3a treatment, indicating that transcription of this gene can no longer be induced by TRP53. As a control, we confirmed the impact of Nutlin-3a treatment, which was able to induce expression of the TRP53 target gene *Puma* in all parental and S63845-resistant cell lines tested, as expected (Supplementary Fig. 3A).

To examine whether the *Bax* gene might be silenced at the epigenetic level in the 560 R2.2 lymphoma cells in which *Bax* transcripts could not be detected, we treated these cells with the hypomethylating agent 5’azacytidine, which inhibits DNMT1, and performed qRT-PCR to look for the restoration of *Bax* mRNA expression levels (Fig. 5C). However, while treatment with 5’azacytidine could induce expression of the known DNMT1-regulated gene *Noxa* in all lymphoma cell lines tested (Supplementary Fig. 3B), no substantial induction of *Bax* mRNA was observed in the 560 R2.2 cells. Therefore, it is possible that *Bax* expression is being prevented by a transcriptional repressor protein or a microRNA in these cells, or by a genetic mutation that we were unable to identify. Interestingly, while 5’azacytidine treatment could increase *Bax* transcript levels in the other drug-resistant BAX KO cell lines tested, it could not restore sensitivity of any of these drug-resistant lymphoma cells to S63845, confirming that a functional BAX protein was not being made in these cells (Fig. 5D).

### Unbiased CRISPR/Cas9 whole genome knockout screens in a human Burkitt lymphoma cell line show that loss of BAX is a major resistance factor for MCL-1-targeting BH3-mimetic drugs

**Fig. 6:**
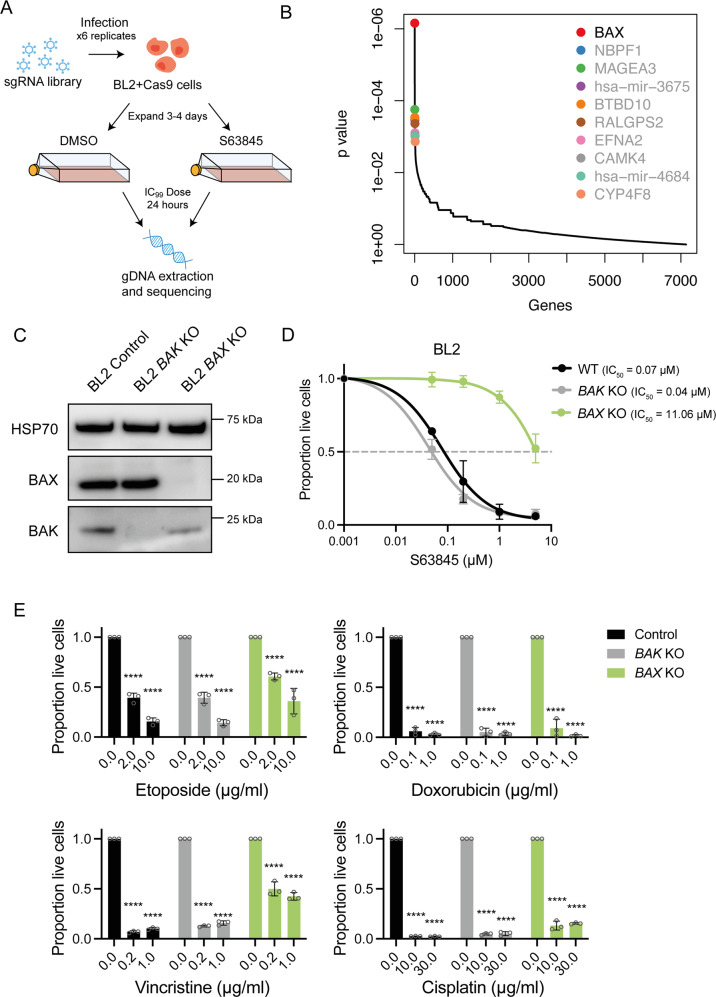
Whole genome CRISPR/Cas9 KO screens reveal loss of BAX as the top factor conferring resistance to BH3-mimetic drugs targeting MCL-1 in human Burkitt lymphoma cells. **A** Schematic of CRISPR/Cas9 KO screen performed in the human Burkitt lymphoma cell line BL2. Cells were treated with DMSO (vehicle, negative control) or 100 nM of S63845 (~IC_99_ dose) for 24 h and surviving cells were sorted by flow cytometry and collected for next-generation sequencing. **B** MAGeCK analysis to identify top enriched sgRNAs in drug-resistant cell populations. Significant hits (FDR < 0.05) are indicated with black text. Loss of BAX was identified as the top factor conferring resistance to S63845 in BL2 cells. **C** Western blot analysis to validate CRISPR/Cas9-mediated loss of BAK or BAX in BL2 cells. Probing for HSP70 was used as a loading control. **D** Dose–response curves for control BL2 cells (containing Cas9 and a non-targeting control sgRNA) and isogenic *BAK* KO or *BAX* KO BL2 lymphoma cells treated with the MCL-1 inhibitor S63845 for 24 h. Live cells were identified as Annexin V/PI double negative by flow cytometry. **E** Isogenic WT, *BAK* KO or *BAX* KO BL2 cells were treated with etoposide, doxorubicin, vincristine or cisplatin for 24 h and cell survival was determined as described above. In (**E**), significance is measured versus the DMSO-treated sample for each cell line. One-way ANOVA was used to determine statistical significance (****p < 0.0001). All data are presented as mean ± SD for 3 independent experiments.

To examine whether our results in the *Eµ-Myc* mouse model could be applied to human cancer cells, we performed a whole genome CRISPR/Cas9 KO screen in the human Burkitt lymphoma cell line BL2 that has wildtype TP53 and is highly sensitive to MCL-1 inhibition [24]. We used the human GeCKO v2 sgRNA library [34], and treated library-infected, Cas9-expressing BL2 cells with either DMSO (vehicle control) or a high dose (IC_99_) of S63845 for 24 h (Fig. 6A). As in our previous screens in the *Eµ-Myc* mouse lymphoma cell lines, loss of *BAX* was identified as the top hit conferring resistance to S63845, whereas sgRNAs targeting *BAK* were not enriched (Fig. 6B).

To validate these results, we used CRISPR/Cas9 to generate isogenic BL2 lymphoma cell lines with *BAK* or *BAX* KO and confirmed the absence of these proteins by western blotting (Fig. 6C). Treatment of these cell lines with S63845 revealed that the *BAX* KO cells were highly resistant, whereas the *BAK* KO cells showed sensitivity similar to the parental cells (Fig. 6D). Of note, single agent treatment with vincristine, doxorubicin, cisplatin and etoposide (all relevant to the treatment of human lymphoma patients [31, 32]) could still kill the *BAX* KO BL2 cells (Fig. 6E), as was observed for the *Eµ-Myc* BAX KO lymphoma cells. This suggests that human MYC-driven lymphomas, with loss of BAX function that arise in response to BH3-mimetic drug treatment, may still respond to conventional chemotherapeutic agents as salvage therapy.

## Discussion

Resistance to BH3-mimetic drugs is a critical and emerging issue which threatens to limit the benefit of this class of anti-cancer therapy. While genetic changes underlying resistance to the BCL-2 inhibitor venetoclax are beginning to be uncovered [5, 23, 26, 35–39], less attention has been focused on resistance to MCL-1 inhibitors, which have entered clinical trials for diverse haematological malignancies [5]. To this end, we sought to identify factors that could confer resistance to MCL-1 inhibitors using murine and human models of highly aggressive, MYC-driven lymphoma.

Our previous work identified TP53 as a critical mediator of the response to MCL-1 and BCL-2 inhibitors in diverse blood cancer cell lines and patient samples [24]. Therefore, we first sought to identify any additional factors which might enable cells lacking TRP53 to escape BH3-mimetic-mediated cell death. Using whole genome CRISPR/Cas9 KO screens, we identified loss of BAX as the top factor conferring resistance of both *Trp53* wildtype and *Trp53*-deficient mouse *Eµ-Myc* lymphoma cell lines to BH3-mimetics targeting MCL-1. Similarly, in a CRISPR/Cas9 KO screen performed in human BL2 cells, loss of BAX was also the top hit. Validation experiments revealed that the IC_50_ for the MCL-1 inhibitor S63845 in cells lacking BAX was >10-fold higher compared to control cells. Consistent with our results, BAX has been identified as a top hit conferring resistance of AML cells to venetoclax in other CRISPR/Cas9 KO screens [25, 26]. Furthermore, AML patient samples exhibiting loss of BAX expression [40], and AML cell lines with CRISPR/Cas9-mediated BAX KO [41] were both found to be resistant to venetoclax. Importantly, in our study, we found that human BL2 and mouse *Eµ-Myc* lymphomas lacking BAX expression could still be killed by both standard-of-care drugs and salvage therapies for lymphoma, indicating that tumours which acquire *BAX* mutations following BH3-mimetic drug treatment may still respond to chemotherapy. These results suggest that the upregulation of pro-apoptotic BH3-only proteins by chemotherapy drugs may act to counter the loss of pro-apoptotic BAX, allowing apoptosis to proceed. While our data are compelling in this regard, further research will be needed to bring these findings to the clinic.

We next wanted to identify resistance-conferring factors that would be likely to occur spontaneously in patients treated with MCL-1 inhibitors. Therefore, we generated *Eµ-Myc* lymphoma cell lines that are resistant to S63845 by culturing them in increasing doses of this drug over time. We identified two major mechanisms driving resistance: loss of BAX expression, and increased expression of pro-survival proteins other than MCL-1. The most prominent was the loss of BAX expression. In two of these cell lines lacking BAX, we identified a splicing mutation likely to result in the preservation of intron 1 in the canonical and most highly expressed mouse *Bax* transcript, *Bax-201*. In a third case, we identified a complete lack of any *Bax* transcription, which could not be restored by treatment with the hypomethylating agent 5’azacytidine, suggesting that this is not due to epigenetic silencing.

The selection via diverse mechanisms to disable BAX expression in these cells illustrates its importance in facilitating cell death in the face of MCL-1 inhibition. Despite these mutations, all *Bax* KO *Eμ-Myc* lymphoma cell lines remained sensitive to killing by diverse chemotherapeutic drugs. Our findings are consistent with recent studies which found that loss of BAX could confer resistance to both venetoclax and S63845 in AMLs [42] and a panel of non-Hodgkin B cell lymphoma cell lines [43]. In addition, in this latter study, low levels of *BAX* mRNA were identified in samples from 3 CLL patients treated with venetoclax, and in CLL cells from another patient it was shown that *BAX* mutations were enriched during venetoclax treatment. Our work complements these findings and reveals that loss of BAX is the most potent resistance factor for MCL-1 inhibition in lymphoma, and also that such resistance can be overcome by treatment with standard-of-care chemotherapeutic drugs. Interestingly, selection for loss of BAX has also been reported in non-malignant myeloid cells of patients undergoing venetoclax therapy for CLL [36].

A major question that arises from our research, and that of the others discussed above, is why the loss of BAX, and not the loss of BAK, is the major mediator of specific resistance to MCL-1 targeting BH3-mimetic drugs. BAX and BAK both function as effectors of apoptosis that, when activated, oligomerise and form pores in the outer mitochondrial membrane. BAX and BAK have largely overlapping functions, best demonstrated by the observation that single KO mice exhibit only minor abnormalities, whereas *Bax/Bak* double KO mice have severe (usually fatal) developmental abnormalities [44, 45]. However, BAX and BAK proteins are found in distinct sub-cellular localisations [46–48]. In addition, they show differential binding to the BCL-2 pro-survival proteins [49–51]. From our earlier studies we do know that both BAX and BAK are activated to some degree after BH3-mimetic drug treatment [24]. Therefore, one hypothesis that could explain our findings is that when a BH3-mimetic drug binds to MCL-1, another pro-survival protein such as BCL-XL preferentially binds to free BAK over BAX, leaving BAX to be the primary mediator of cell death in this context. Another possibility is that the MCL-1 targeting BH3-mimetic S63845 preferentially inhibits BAX over BAK from binding to MCL-1. These different scenarios are difficult to measure because of the different states in which BAX and BAK can exist within cells, including (inert) monomers, homodimers, higher-order oligomers, as well as in complexes with different pro-survival proteins. The future generation of antibodies that can distinguish between these states, as well as affinity measurements for the full-length proteins in a mitochondrial context, is essential to interrogate this with greater precision.

The second major mechanism driving resistance we identified in our S63845-resistant cells was upregulation of the pro-survival proteins BCL-XL and A1. Many studies have reported the over-expression of pro-survival BCL-2 proteins as resistance factors for venetoclax therapy, including in patient samples and in in vitro whole genome CRISPR/Cas9 screens [35, 37, 39, 52, 53]. In our study, *Eµ-Myc* lymphoma cells that had achieved resistance to S63845 through upregulation of BCL-XL and A1 also showed resistance to etoposide treatment due to the over-expression of these pro-survival proteins preventing apoptosis. However, the lymphoma cells with upregulation of BCL-XL/A1 could be re-sensitised to apoptosis by co-inhibition of BCL-XL alongside MCL-1, illustrating that inhibition of compensatory, non-targeted pro-survival BCL-2 proteins is an effective therapeutic strategy to kill malignant cells that have become resistant to a particular BH3-mimetic drug. However, it must be noted that the combination of MCL-1 and BCL-XL targeting BH3-mimetics is predicted to be toxic to healthy cells [54]. Therefore, these therapeutic strategies are likely to only be possible in patients when such drugs can be targeted to the cancer cells directly through, for example, antibody conjugation.

Understanding the factors which can guard against cell killing by BH3-mimetic drugs is the first step in enabling the long-term success of these new anti-cancer therapies. In this study, we have identified factors (loss of BAX; gain of BCL-XL and/or A1) that confer resistance to the new class of BH3-mimetics targeting MCL-1, drugs which are currently being evaluated in clinical trials for diverse blood cancers. Furthermore, we identified therapeutic strategies to overcome resistance based on an understanding of underlying gene expression changes, including combining MCL-1 targeting BH3-mimetic drugs with conventional chemotherapeutic drugs (e.g., etoposide, vincristine, doxorubicin or cisplatin) or with BH3-mimetics that target other pro-survival proteins. This work illustrates how mechanistic studies can inform new therapeutic strategies to overcome resistance and therefore enable durable responses to BH3-mimetic drugs in the clinic.

## Materials and methods

### Cell lines

Mouse *Eµ-Myc* lymphoma-derived cell lines AH15A, AF47A and 560 were cultured in high glucose Dulbecco’s modified Eagle’s medium (DMEM) containing 10% heat-inactivated foetal bovine serum (FBS; Sigma #F9423), 100 µM asparagine (Sigma #A4284), 50 µM β-mercaptoethanol (Sigma #M3148), 100 U/mL penicillin and 100 µg/mL streptomycin (Gibco #15140122). *Eµ-Myc* lymphoma cells were maintained in a humidified incubator at 37 °C with 10% CO_2_. The human Burkitt lymphoma cell line BL2 was a kind gift from Prof Alan Rickinson, The University of Birmingham, UK. BL2 cells were authenticated by STR profiling at the Australian Genome Research Facility and maintained in RPMI-1640 medium containing 10% heat-inactivated FBS, 1 mM sodium pyruvate (Gibco #11360070), 2 mM L-glutamine (Gibco #25030081), 50 μM α-thioglycerol (Sigma #M-6145), 100 U/mL penicillin and 100 µg/mL streptomycin in a humidified incubator at 37 °C with 5% CO_2_. HEK293T cells were cultured in DMEM containing 10% heat-inactivated FBS, 100 U/mL penicillin and 100 µg/mL streptomycin, and maintained in a humified incubator at 37 °C with 10% CO_2_. Mycoplasma screening of all cell lines used was conducted regularly (MycoAlert kit; Lonza #LT07-118).

### CRISPR/Cas9 whole genome knockout screening

*Eµ-Myc* and BL2 cell lines were stably transduced with lentiviruses carrying FuCas9-Cherry (Addgene Plasmid #70182) using established methods [15]. Lentiviral supernatant was used to transduce 1 × 10^5^ *Eµ-Myc* or BL2 cells by centrifugation at 2200 rpm for 2 h at 32 °C in the presence of polybrene (made in house, final concentration 8 µg/mL). After 72 h, mCherry-positive cells were sorted on a FACSAria Fusion flow cytometer (BD Biosciences). Three to six replicates of 3 × 10^5^ Cas9-expressing lymphoma cells were then transduced with the whole genome CRISPR knockout libraries, either the YUSA library for mouse *Eµ-Myc* lymphoma cells (87,897 sgRNAs targeting 19,150 protein-coding genes) [30] or the GeCKO v2 library for human BL2 cells (123,411 sgRNAs targeting 19,050 protein-coding genes) [34]. Cells were expanded for 2–3 days and then each replicate was split into two flasks of 7 × 10^6^ cells each. For short-term CRISPR/Cas9 screens (*Trp53* KO *Eµ-Myc* cell lines and BL2 cells), one flask was treated for 24 h with DMSO (vehicle control) and the other with a high dose of S63845 that kills 99% of parental lymphoma cells (IC_99_; 1 µM for *Trp53* KO *Eµ-Myc* cell lines, 100 nM for BL2 cells), followed by flow sorting of live, drug-resistant cells. For longer-term screens (wildtype *Eµ-Myc* lymphoma cell lines), cells were treated with DMSO (vehicle control) or with suboptimal (100 nM = ≤ IC_50_) doses of S63845 every 2–3 days over a 2-week period. Cell pellets were collected and genomic DNA extracted using the DNeasy Blood and Tissue Kit (QIAGEN # 69506). DNA was amplified using library-specific primers with indexing overhangs allowing for Illumina sequencing. Samples were pooled and sequenced on the Illumina NextSeq 550. MAGeCK v0.5.9.4 [55] was used to map reads to sgRNAs and quantify sgRNA enrichment. For all CRISPR/Cas9 screens, genes with FDR < 0.05 were considered to be significant hits.

### Targeted CRISPR/Cas9 gene editing

CRISPR/Cas9 gene editing was employed to generate *Trp53*, *Bak* or *Bax* single KO, and *Bak/Bax* double KO mouse *Eµ-Myc* and/or human BL2 lymphoma cell lines. Cells harbouring a non-targeting sgRNA were also generated and used as ‘control’ cells in all experiments using CRISPR/Cas9-dervied cell lines. Lentiviruses containing sgRNAs in the doxycycline-inducible FgH1tUTG backbone (Addgene Plasmid #70183) were produced and cells were transduced with these vectors using established methods [15]. Successfully transduced GFP-positive cells were sorted as described above. sgRNA sequences used are listed in Supplementary Table 1. To induce sgRNA expression, culture medium was supplemented with 1 µg/mL doxycycline hyclate (Sigma #D9891) each day for 5 days. Efficient knockout of target proteins was confirmed by western blotting.

### Western blotting

Cell pellets were resuspended in radioimmunoprecipitation assay (RIPA) buffer containing protease inhibitors (Roche #11836145001) and incubated on ice for 25 min. Centrifugation was performed at 13,300 rpm for 10 min at 4 °C and supernatant containing proteins was collected. Protein levels were quantified using the Pierce BCA Protein Assay Kit (Thermo Fisher, #23225) and 10 µg was run on a 10% NuPAGE Bis-Tris 1.5 mm gel in 2-(N-morpholino)-ethanesulfonic acid (MES) running buffer. Proteins were transferred onto nitrocellulose membranes using the iBlot2 (Thermo Fisher). Membranes were blocked at room temperature (RT) for 1 h in phosphate-buffered saline containing 0.1% Tween-20 (PBST) and 5% skim milk. Membranes were rinsed in PBST and incubated overnight at 4 °C in primary antibody diluted in PBST containing 5% bovine serum albumin. The next day, membranes were washed in PBST and incubated for 1 h at RT in HRP-conjugated secondary antibodies diluted in PBST containing 5% skim milk. Membranes were washed again in PBST and imaged by the addition of Immobilon Forte (Merck Millipore #WBLUF0500) on a ChemiDoc system (Bio-Rad). Comparison to Precision Plus Protein Dual Color Standard (Bio-Rad #1610374) was used to estimate protein weight. Antibodies used for western blotting are listed in Supplementary Table 2. Uncropped blots are shown in the supplementary “original data” file 2.

### Apoptosis assays

Cells were plated at 3 × 10^4^ cells/well of a flat-bottom 96-well plate in triplicate and drugs were added to give the indicated final concentrations. The following drugs were used: S63845 (MCL-1 inhibitor; Active Biochem #A-6044), A-1331852 (BCL-XL inhibitor; AbbVie, provided by Dr G. Lessene, WEHI), ABT-199 (BCL-2 inhibitor; Active Biochem #A-1231), ABT-737 (BCL-2, BCL-XL and BCL-W inhibitor; Active Biochem #A-1002), etoposide (Ebewe Interpharma), ionomycin (Sigma-Aldrich #I9657), cisplatin (Hospira Australia), doxorubicin (Ebewe Interpharma), vincristine (Sigma-Aldrich #V8879), Nutlin-3a (Cayman Chemicals #18585) and 5’azacytidine (Sigma-Aldrich #A2385). Cells were incubated with the drugs at 37 °C for 24 h. Plates were centrifuged and cells stained in Annexin V binding buffer (10 mM HEPES pH 7.4, 140 mM NaCl, 2.5 mM CaCl_2_) containing Annexin V-Alexa Fluor 647 (1:1000, made in house) and 1 µg/mL propidium iodide (PI; Sigma-Aldrich #P4170). Viable cells (Annexin V and PI double negative) were quantified on an LSR II W flow cytometer (BD Biosciences) and data were analysed in FlowJo v10.8 (BD Life Sciences). For each sample, the proportion of live cells was normalised to the DMSO-treated (vehicle, control) sample for each cell line. Non-linear regression (curve fit) analysis was used to generate dose–response curves and calculate IC_50_ values in GraphPad Prism v9. All data are presented as mean ± standard deviation of 2–3 independent experiments. One-way ANOVA with Dunnett’s post hoc test was used to compare control and treated or parental and drug-resistant groups.

### Derivation of *Eµ-Myc/Bax* KO and *Eµ-Myc/Bak* KO lymphoma cell lines

All experiments involving animals were performed in accordance with the guidelines set out by the WEHI Animal Ethics Committee. All mice were on a C57BL/6 background and bred at WEHI. Mice deficient for BAX [56] or BAK [44] were crossed with *Eµ-Myc* mice [17]. Offspring were monitored for lymphoma and euthanised when they reached the ethical endpoint as determined by trained animal technicians according to the WEHI Animal Ethics Committee guidelines. Enlarged organs (spleen, lymph nodes, thymus) were harvested, homogenised and filtered through a 100 µm strainer to generate a single cell suspension. *Eµ-Myc*/*Bax*^*−/−*^ (*Bax* KO) and *Eµ-Myc*/*Bak*^*−/−*^ (*Bak* KO) lymphoma cell lines were cultured as described above.

### Generation of MCL-1-targeting BH3-mimetic drug-resistant lymphoma cell lines

To generate drug-resistant *Eµ-Myc* lymphoma cell lines, cells were cultured in increasing doses of S63845 over time. Cells were allowed to recover to >70% viability before higher doses of the drug were added. Cells were cultured until they could withstand doses of S63845 10–50× the IC_50_ concentrations of the parental cell lines. Two independent culturing campaigns were undertaken. For each campaign, cell lines were generated that could resist 1 µM S63845 (resistant cell lines 1.1 and 2.1). These cell lines were then cultured further in increasing doses of S63845 until they could withstand 5 µM S63845 (resistant cell lines 1.2 and 2.2). Resistance to S63845 was confirmed by short-term apoptosis assays as described above.

### Next-generation sequencing of mouse *Bax* gene coding regions

Genomic DNA was isolated from parental and S63845-resistant cells using the DNeasy Blood and Tissue Kit (QIAGEN). Primers tiling the murine *Bax* promoter and exon sequences were designed using Primer Blast and modified with overhangs to allow for the indexing of products for sequencing on the Illumina platform. Primer sequences are listed in Supplementary Table 3. For each primer pair, 200 ng of genomic DNA was PCR amplified using GoTaq Green Mix (Promega #M712) along with 0.25 µM of each primer. The following cycling conditions were used: denaturation at 95 °C for 3 min, followed by 35 cycles of 95 °C for 30 s, 60 °C for 30 s and 72 °C for 30 s. The final extension was performed at 72 °C for 5 min. Products were pooled and cleaned up using AmPure XP beads (Beckman Coulter #A63880) and sequenced on an Illumina MiSeq. Sequencing reads from S63845-resistant cells were aligned to reads from parental cells using the R package DECIPHER [57] in order to identify mutations in *Bax* coding regions.

### Quantitative reverse-transcriptase PCR (qRT-PCR)

To obtain intact cells for qRT-PCR, cells were incubated with 25 μM of the broad-spectrum caspase inhibitor Q-VD-OPh (MedChem Express # HY-12305) for 15 min prior to treatment with 5’azacytidine or Nutlin-3a. After 24 h, cell pellets were collected and resuspended in 0.5 mL TRIzol reagent (Thermo Fisher #15596026). RNA was isolated according to the manufacturer’s instructions. cDNA was prepared using the Superscript III First Strand Synthesis System (Thermo Fisher #18080051) according to the manufacturer’s instructions. qRT-PCR reactions were performed using TaqMan Fast Advanced Master Mix (Thermo Fisher, #4444963) according to the manufacturer’s instructions. TaqMan probes to detect mouse *Puma/Bbc3* (Mm00519268_m1), *Noxa/Pmaip1* (Mm00451763_m1), *Bax* (Mm00432050_m1) and *Hmbs* (Mm01143545_m1) were used (Thermo Fisher). Reactions were run on a Quantstudio 12 K Flex Real-Time PCR System (Thermo Fisher). Cycle threshold (Ct) values for each gene were normalised to the housekeeping control gene (*Hmbs*) Ct value for that sample and all data are presented relative to the DMSO-treated parental cell line. All qRT-PCR data are presented as mean ± standard deviation of two independent experiments.

## Supplementary information

Original Data File

Supplementary Material

Pre-authorship form

checklist

author contribution form

## Data Availability

Data are available upon request to Gemma L. Kelly (gkelly@wehi.edu.au).
