# Supplementary material for: Lymphoma cells lacking pro-apoptotic BAX are highly resistant to BH3-mimetics targeting pro-survival MCL-1 but retain sensitivity to conventional DNA-damaging drugs
Source: Cell Death Differ. 2023 Feb 8;30(4):1005–17. doi: 10.1038/s41418-023-01117-0 (PMC10070326; doi:10.1038/s41418-023-01117-0)

Uncropped blots for Figure 1A

TRP53

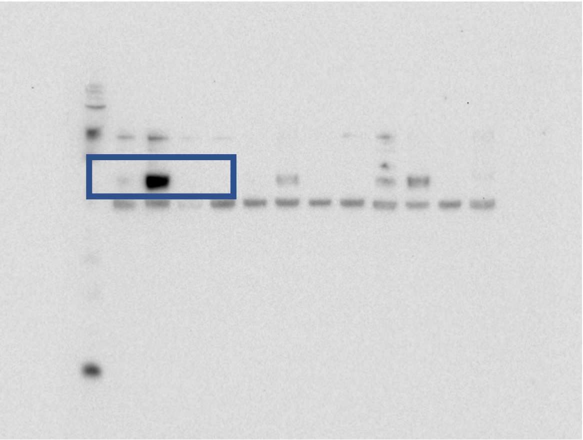

β-ACTIN

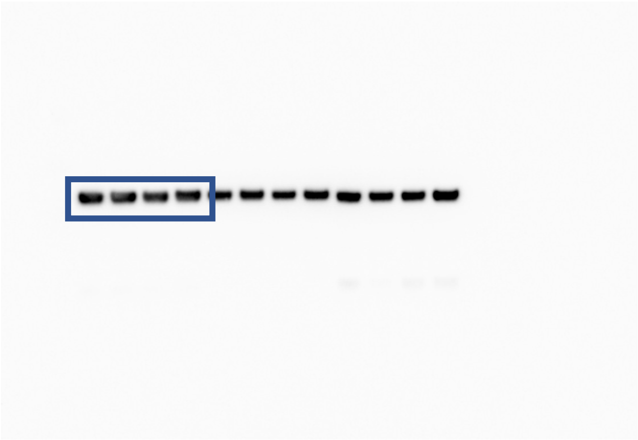

Uncropped blots for Figure 2C

HSP70

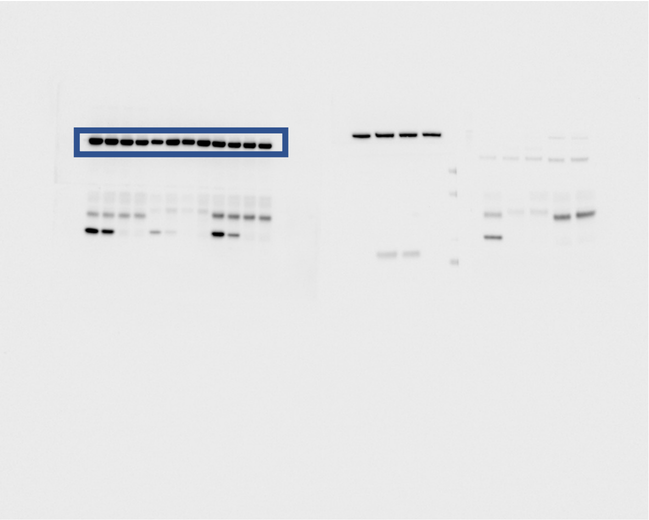

BAX

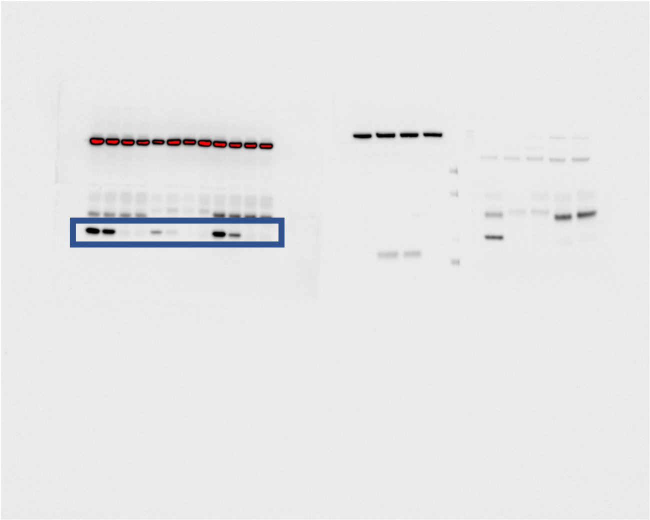

BAK

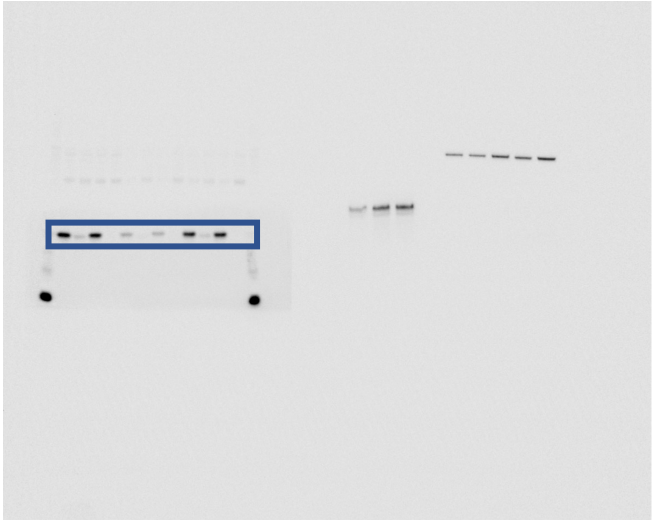

TRP53

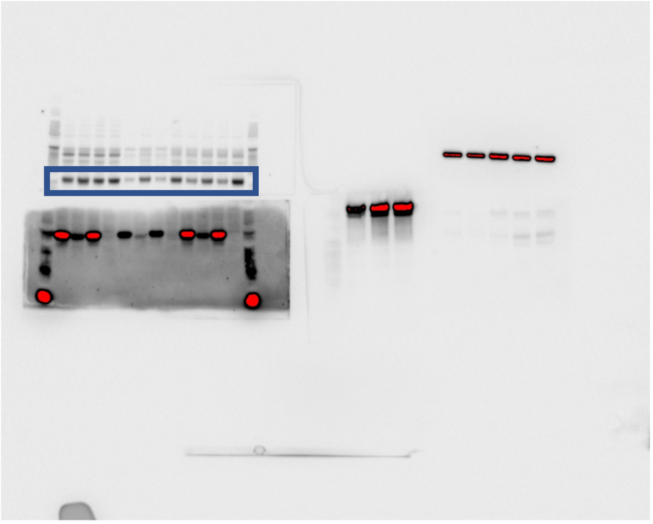

Uncropped blots for Figure 3C

HSP70

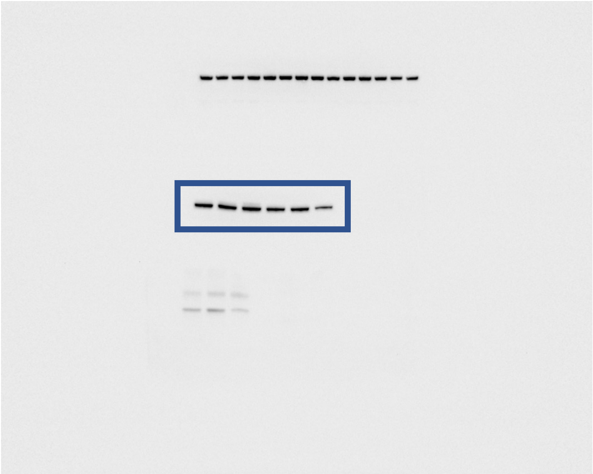

BAX

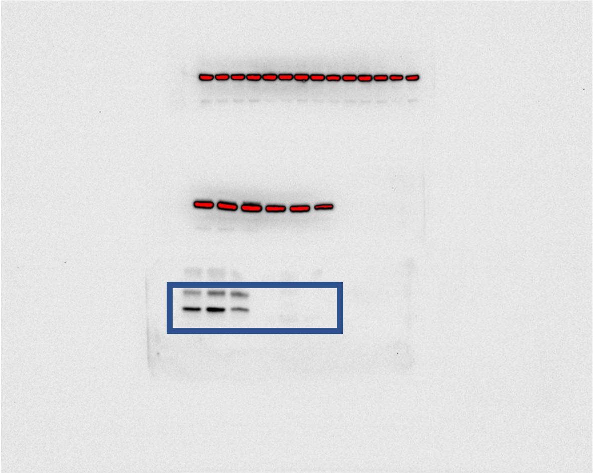

BAK

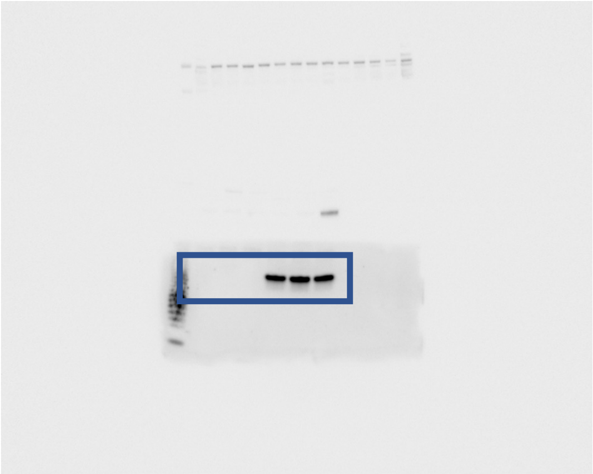

TRP53

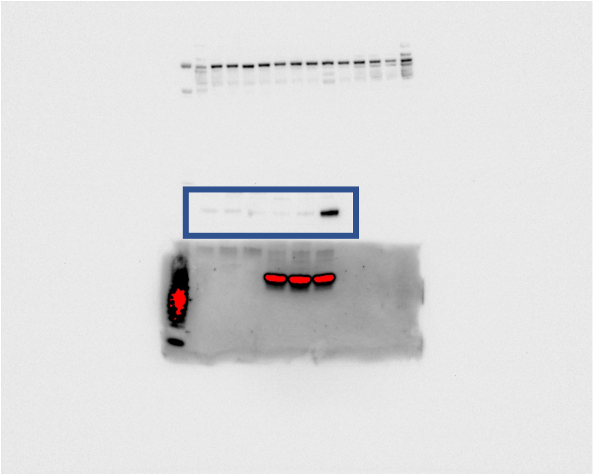

Uncropped blots for Figure 4A

BAK

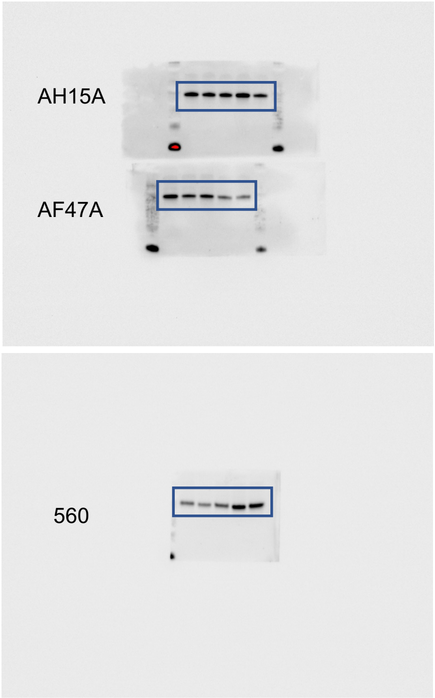

BAX

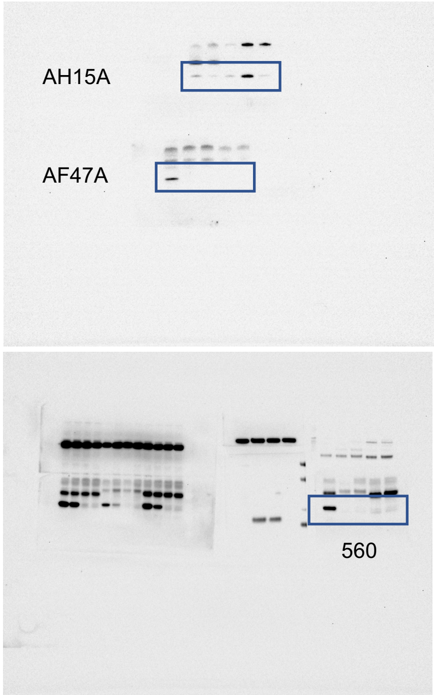

TRP53

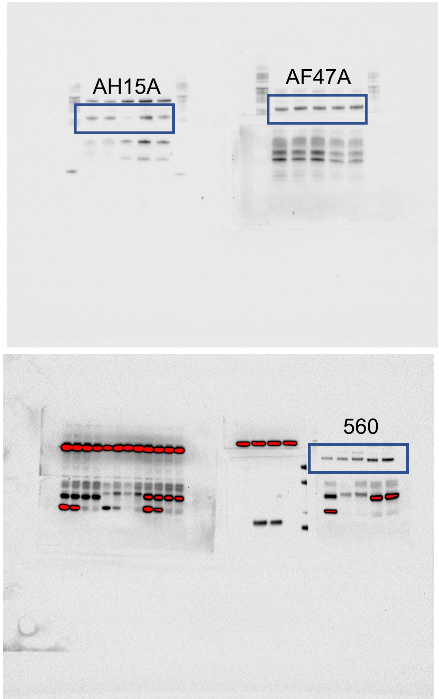

HSP70

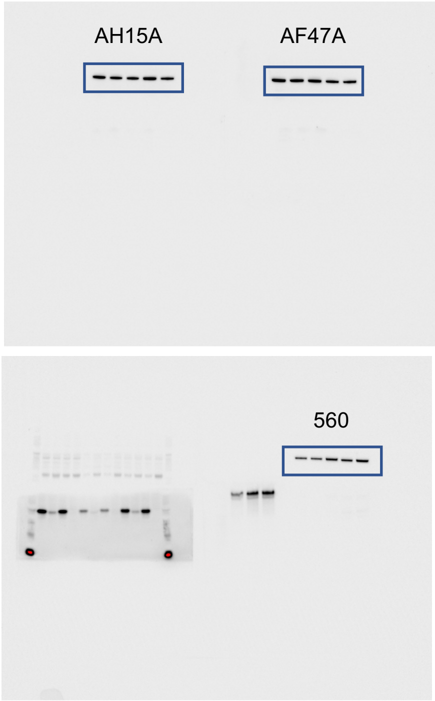

BCL-2

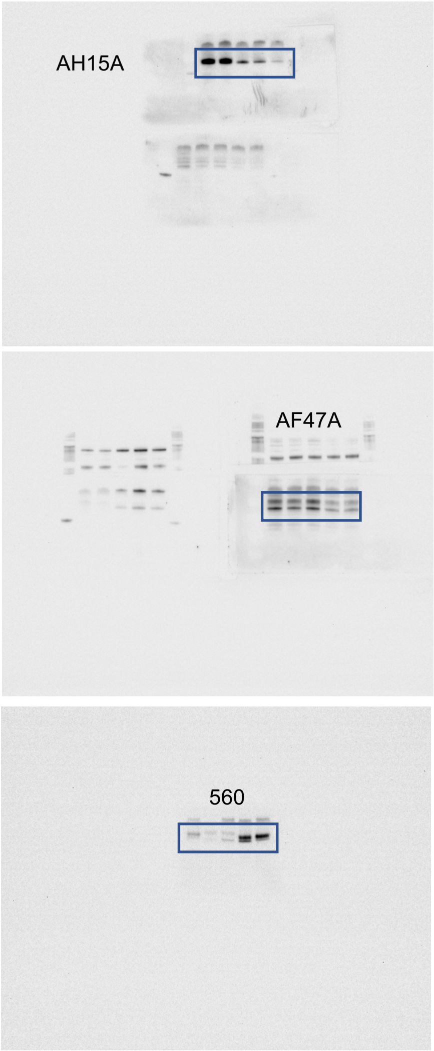

BCL-XL

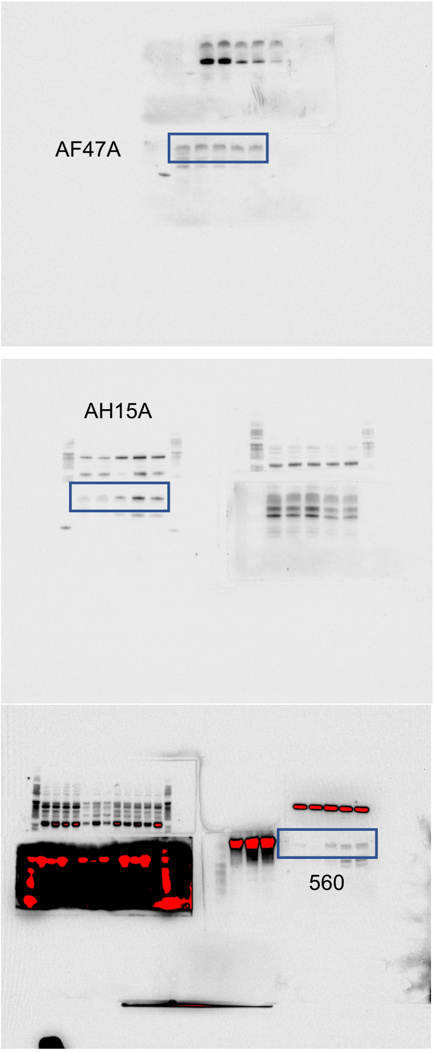

Uncropped blots for Figure 4A

HSP70*

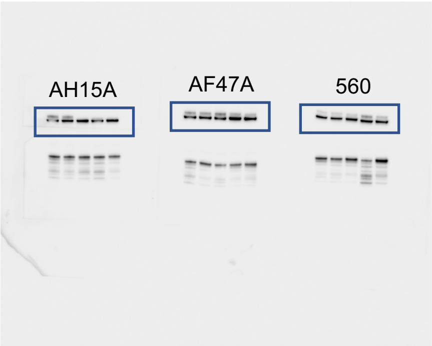

A1

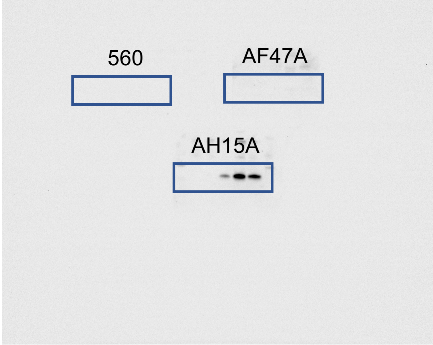

MCL-1

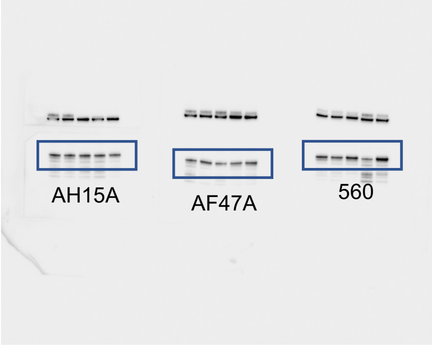

*Lysates were rerun on a fresh gel for probing with these antibodies. This repeated HSP70 loading control was consistent with the one from the previous gel which is included in the manuscript, therefore this one was not shown in the manuscript.

BIM

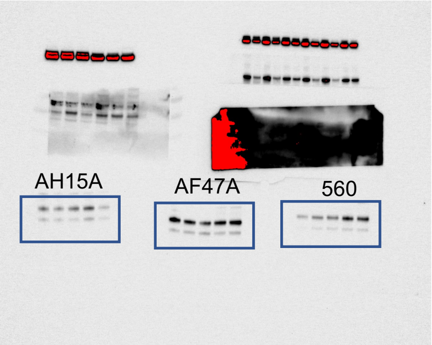

PUMA

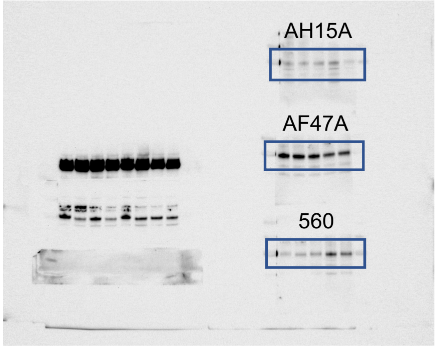

Uncropped blots for Figure 6C

HSP70

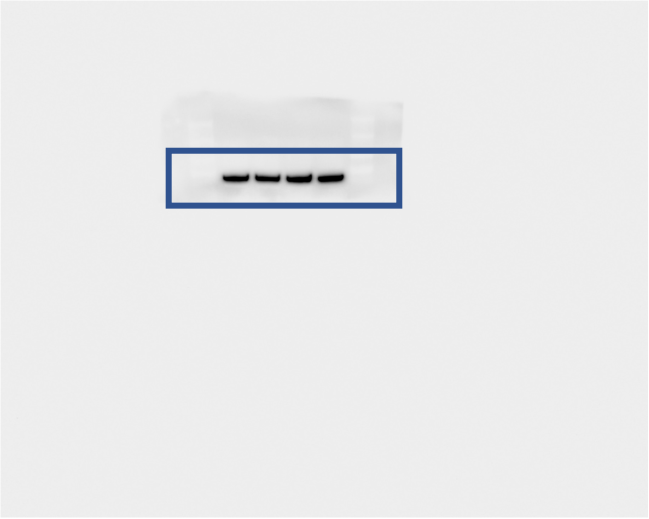

BAX

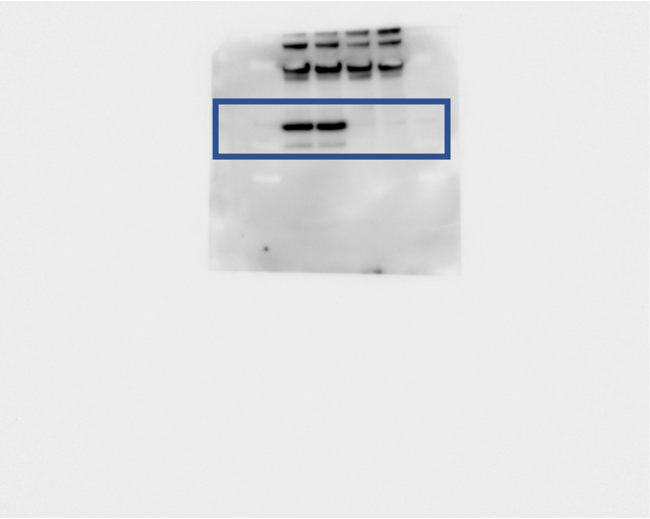

BAK

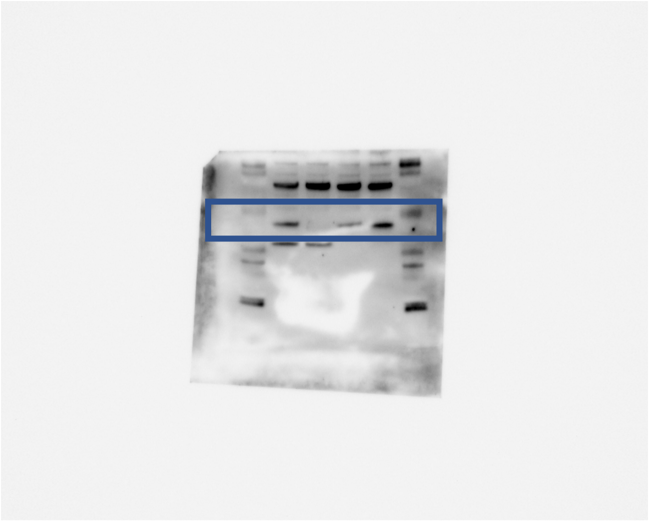

Supplement: Supplementary file 1 — Original Data File [file 41418_2023_1117_MOESM1_ESM.pdf]
