# Supplementary material for: Lymphoma cells lacking pro-apoptotic BAX are highly resistant to BH3-mimetics targeting pro-survival MCL-1 but retain sensitivity to conventional DNA-damaging drugs
Source: Cell Death Differ. 2023 Feb 8;30(4):1005–17. doi: 10.1038/s41418-023-01117-0 (PMC10070326; doi:10.1038/s41418-023-01117-0)
Supplement: Supplementary file 2 — Supplementary Material [file 41418_2023_1117_MOESM2_ESM.docx]

**Supplementary Table 1.** Sequences of sgRNAs used in this study.

| **Target gene** | **Target exon** | **sgRNA sequence 5’-3’** |
| --- | --- | --- |
| Mouse *Bim* (non-targeting negative control for human cells) | 3 | GCACAGGAGCTGCGGCGGAT |
| Mouse *Trp53* | 5 | GAGCGCTGCTCCGATGGTGA |
| Mouse *Bak* | 1 | TCCATCTCGGGGTTGGCAG |
| Mouse *Bax* | 2 | CAGTTCATCTCCAATTCGC |
| Human *BIM* (non-targeting negative control for mouse cells) | 3 | GCCCAAGAGTTGCGGCGTAT |
| Human *BAK* | 5 | GGCCATGCTGGTAGACGTG |
| Human *BAX* | 4 | TCTGACGGCAACTTCAACTG |

**Supplementary Table 2.** Antibodies used for western blotting.

| **Target** | **Clone** | **Dilution** | **Species raised in** | **Source** |
| --- | --- | --- | --- | --- |
| **Primary antibodies** | | | | |
| BAK | polyclonal | 1:2000 | Rabbit | Sigma #B5897 |
| Mouse BAX | 5B7 | 1:2000 | Mouse | Sigma #B9054 |
| Mouse TRP53 | CM5 | 1:2000 | Rabbit | Novocastra, #NCL-p53-CM5p |
| MCL-1 | 19C4-15 | 1:2000 | Rat | WEHI mAb Facility |
| BCL-2 | 7/BCL-2 | 1:2000 | Mouse | BD Biosciences #610539 |
| BCL-XL | 9C9 | 1:1000 | Rat | WEHI mAb Facility |
| Mouse A1 | 6D6 | 1:100 | Rat | WEHI mAb Facility |
| PUMA | polyclonal | 1:500 | Rabbit | Prosci #3043 |
| BIM | polyclonal | 1:1000 | Rabbit | Enzo Life Sciences, #ADI-AAP-330-E |
| Human BAX | 21C10-23-8-38-P | 1:1000 | Rat | WEHI mAb Facility |
| HSP70 | N6 | 1:10000 | Mouse | Dr. R. Anderson, Olivia Newton John Cancer Centre, Melbourne, Australia |
| β-ACTIN | AC-74 | 1:5000 | Mouse | Sigma #A2228 |
| **Secondary antibodies** | | | | |
| Mouse IgG | N/A | 1:2000 | Goat | Southern Biotech, #1010-05 |
| Rat IgG | N/A | 1:5000 | Goat | Southern Biotech, #3010-05 |
| Rabbit IgG | N/A | 1:5000 | Goat | Southern Biotech, #4010-05 |

**Supplementary Table 3.**  Sequencing primers for the analysis of the mouse *Bax* promoter and exons.

| **Primer Pair** | **FWD sequence (5’-3’)** | **REV sequence (5’-3’)** |
| --- | --- | --- |
| 1 | CTGCAGCGAGCGATGATGA | CCTAGTCCTCCGCCTCCC |
| 2 | ACGTGACCGTGGTGCGCCG | CGCACGCGGATGTCCGCTCT |
| 3 | ACCCCGAGAGCGGACATC | GCCTGGCTACTGCTTCTGAT |
| 4 | GTTTGCTGTGGAGCTGGGAT | ATCTGTTCAGAGCTGGTGGG |
| 5 | TTTCCTCCTCTCTCCCCCAG | CTGCTCCAAGGTCAGCTCAG |
| 6 | GCTTGGTTCTCAACATTCTGCT | GAGACCCCTTGACGGACCC |
| 7 | CATCTCACCCAGCGTCTGTT | TCTGTTCCCTGGTCCTCACA |
| 8 | GCAAGGGCTTGAGATCACCT | ATAGCTAGGCCTGAGGGGTC |
| 9 | CAGGGTGGCTGGGTGAGA | GGTTGACCAGAGTGCGTAGG |
| 10 | TGGTATTGAAGTCACACCCATC | CCCATTCATCCCAGGAAAATGTC |
| 11 | CCTTGGACTGTGTCTTTTCTTCAT | GAGCGGGTCCCTGATTGGA |
| 12 | AGCCTCTCAATGCTGGGATG | ACGTGATCATCATCGCTCGC |
| 13 | AGGTATGGACCATCACTCTGGT | AGATTCATGGTAGAGAGCACTAAGG |
| 14 | CACAGGCATCTGCTTGCTTG | GATTGCTCCAATCTGGGGAG |

**Supplementary Figure 1**


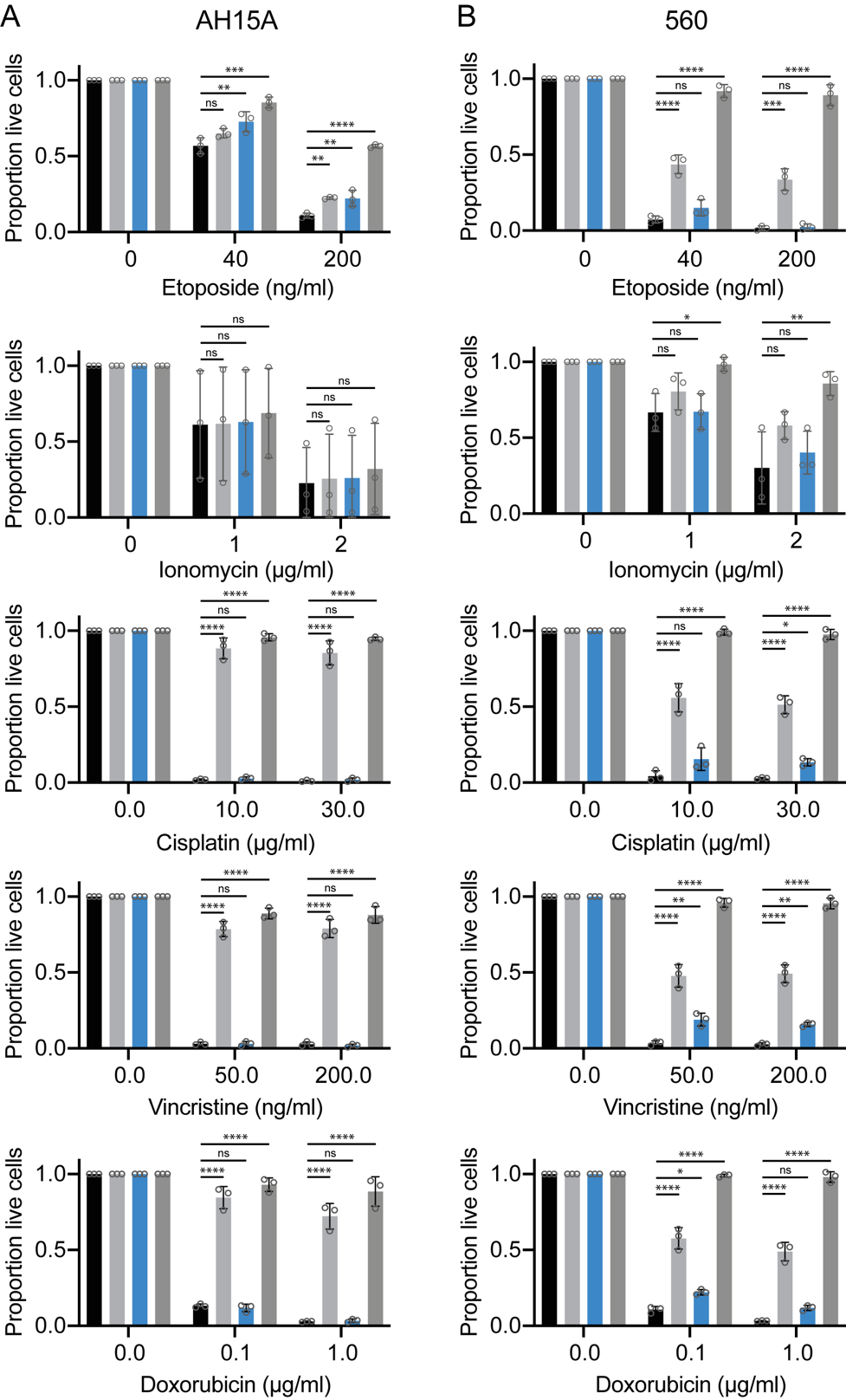


**Supplementary Fig 1. *Eµ-Myc* lymphoma cells lacking BAX retain sensitivity to cytotoxic agents.** Isogenic WT, *Bak* KO, *Bax* KO or *Bak*/*Bax* double KO AH15A and 560 mouse *Eµ-Myc* lymphoma cells treated with the cytotoxic drugs doxorubicin, vincristine, cisplatin, etoposide or ionomycin for 24 h. Live cells were identified as Annexin V/PI double negative by flow cytometry. All data are presented as mean ± S.D. for 3 independent experiments. One way ANOVA was used to measure statistical significance (**p<0.01, ***p<0.001, ****p<0.0001, ns=not significant).

**Supplementary Figure 2**

**
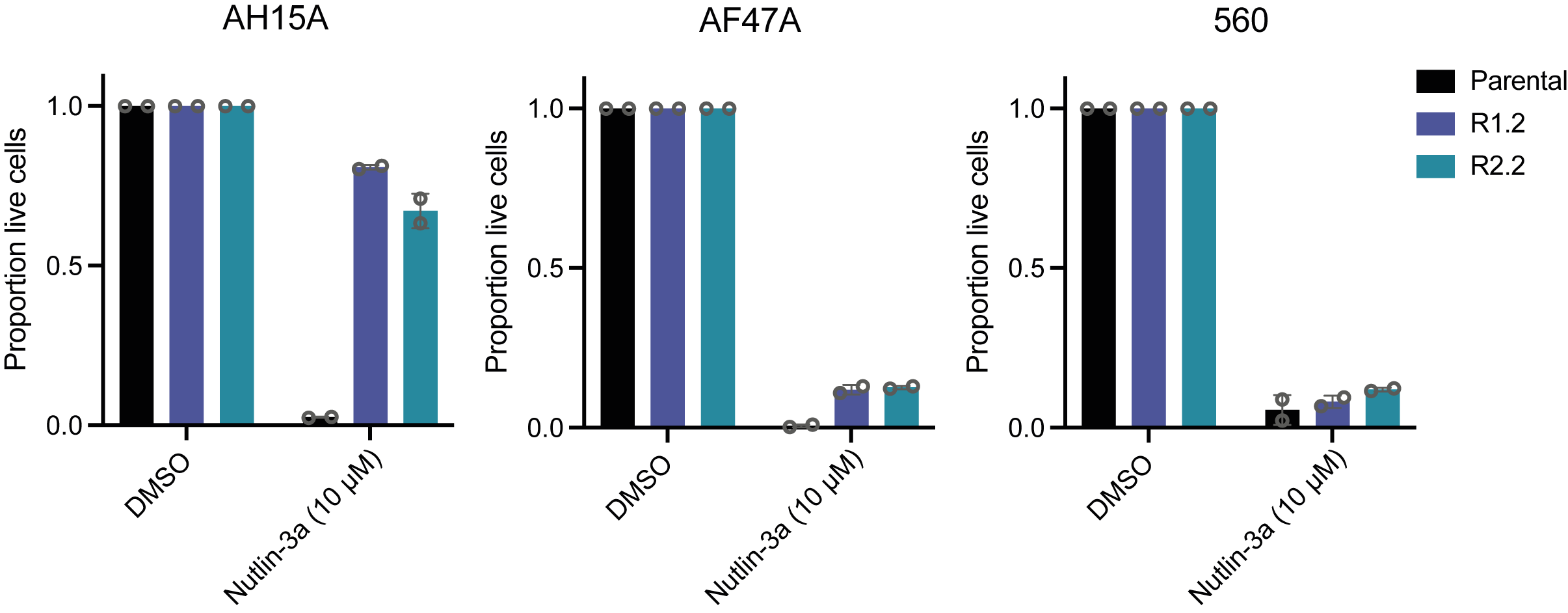
**

**Supplementary Fig 2. Sensitivity of S63845-resistant cells to Nutlin-3a.** Parental AH15A, AF47A and 560 cells and their derived S63845-resistant variants were treated with 10 µM Nutlin-3a for 24 h and live cells quantified by Annexin V/PI staining. All data are presented as mean ± S.D. for 2 independent experiments.

**Supplementary Figure 3**


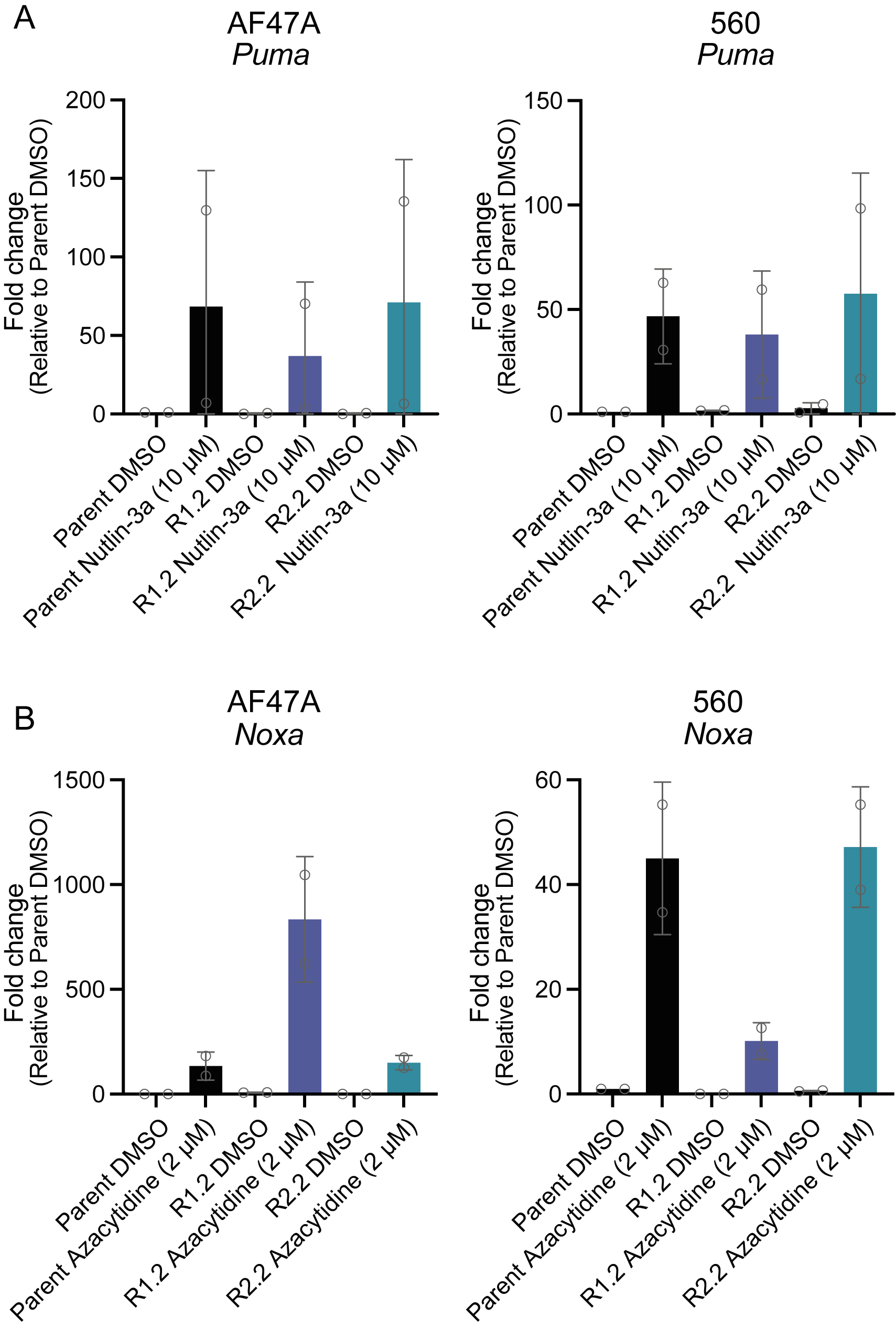


**Supplementary Fig 3. qRT-PCR for *Puma* and *Noxa* to validate impact of Nutlin-3a and 5’azacytidine, respectively.** (A) qRT-PCR of parental and drug resistant *Eµ-Myc* lymphoma cells treated with DMSO (vehicle, negative control) or the TRP53 activating drug Nutlin-3a for 24 h. *Puma* is a direct transcriptional target of TRP53. Data are shown normalised to the housekeeping gene *Hmbs*, and relative to the parental *Eµ-Myc* lymphoma cell line treated with DMSO (vehicle, negative control). (C) qRT-PCR of parental and drug resistant *Eµ-Myc* lymphoma cells treated for 24 h with DMSO or the hypomethylating agent 5’azacytidine (inhibitor of DNMT1). *Noxa* is transcriptionally regulated by DNMT1. Data are shown normalised to the housekeeping gene *Hmbs*, and relative to the parental cell line treated with DMSO.­ Data are presented as mean ± S.D. for 2 independent experiments.
