# Supplementary material for: Lymphoma cells lacking pro-apoptotic BAX are highly resistant to BH3-mimetics targeting pro-survival MCL-1 but retain sensitivity to conventional DNA-damaging drugs
Source: Cell Death Differ. 2023 Feb 8;30(4):1005–17. doi: 10.1038/s41418-023-01117-0 (PMC10070326; doi:10.1038/s41418-023-01117-0)
Supplement: Supplementary file 3 — Pre-authorship form [file 41418_2023_1117_MOESM3_ESM.pdf]

SPRINGER NATURE

Change of authorship request form - Journals (pre-acceptance)

Important information. Please read.

- This form should be used by authors to request any change in authorship (adding/deleting authors) including changes in corresponding authors. This form should not be used for name changes. Please fully complete all sections. Use black ink and block capitals and provide each author's full name with the given name first followed by the family name.

- By signing this declaration, all authors guarantee that the order of the authors are in accordance with their scientific contribution, if applicable as different conventions apply per discipline, and that only authors have been added who made a meaningful contribution to the work.

- Please note, in author collaborations where there is formal agreement for representing the collaboration, it is sufficient for the representative or legal guarantor (usually the corresponding author) to complete and sign the Authorship Change Form on behalf of all authors, **next to the added/removed author(s)**. **(Complete Section 3, followed by Section 6.)**
  In author collaborations where there is no formal agreement for representing the collaboration and **there are more than 10 authors**, one may sign for all, provided the signer appends correspondence that attests that each of the authors have agreed to the change **and the added/removed authors sign the form**. **(Complete Section 3, followed by Section 6.)**

- Please note, we cannot investigate or mediate any authorship disputes. If you are unable to obtain agreement from all authors (including those who you wish to be removed) you must refer the matter to your institution(s) for investigation. Please inform us if you need to do this.

- If you are not able to return a fully completed form within **30 days** of the date that it was sent to the author requesting the change, we may have to withdraw your manuscript. We cannot publish manuscripts where authorship has not been agreed by all authors (including those who have been removed).

- Incomplete forms will be rejected.

- Please return/upload this form, fully completed, to the Journals Editorial Office. The Journal and/or Publisher will consider the information you have provided to decide whether to approve the proposed change in authorship. We may decide to contact your institution for more information or undertake a further investigation, if appropriate, before making a final decision.

SPRINGER NATURE Springer Nature is one of the world's leading global research, educational and professional publishers, created in May 2015 through the combination of Nature Publishing Group, Palgrave Macmillan, Macmillan Education and Springer Science+Business Media.

SPRINGER NATURE

Change of authorship request form - Journals (pre-acceptance)

Section 1: Please provide the current title of manuscript

Manuscript ID no.: CDD-22-1867

Title:
Lymphoma cells lacking pro-apoptotic BAX are highly resistant to BH3-mimetics targeting pro-survival MCL-1 but retain sensitivity to conventional DNA damaging drugs

Section 2: Please provide the previous authorship, in the order shown on the manuscript before the changes were introduced. Please indicate the corresponding author by adding (CA) behind the name.

	First name(s)	Family name	ORCID or SCOPUS id, if available
1st author	Sarah	Diepstraten	
2nd author	Savannah	Young	
3rd author	John	La Marca	
4th author	Zilu	Wang	
5th author	Andreas	Strasser	
6th author	Gemma	Kelly (CA)	
7th author			
8th author			
9th author			
10th author			

Please use an additional sheet if there are more than 10 authors.

SPRINGER NATURE Springer Nature is one of the world's leading global research, educational and professional publishers, created in May 2015 through the combination of Nature Publishing Group, Palgrave Macmillan, Macmillan Education and Springer Science+Business Media.

SPRINGER NATURE

Change of authorship request form - Journals (pre-acceptance)

Section 3: Please provide a justification for change. Please use this section to explain your reasons for changing the authorship of your manuscript, e.g. what necessitated the change in authorship? Please refer to the (journal) policy pages for more information about authorship. Please explain why omitted authors were not originally included and/or why authors were removed on the submitted manuscript.

We wish to add Ruth Kluck as an author as she contributed advice and reagents to the new experiments performed to satisfy the reviewer comments, as well as critically edited the manuscript for resubmission.

Section 4: Proposed new authorship. Please provide your new authorship list in the order you would like it to appear on the manuscript. Please indicate the corresponding author by adding (CA) behind the name. If the Corresponding Author has changed, please indicate the reason under section 3.

	First name(s)	Family name (this name will appear in full on the final publication and will be searchable in various abstract and indexing databases)	Affiliated institute	E-mail address
1st author	Sarah	Diepstraten	WEHI, Melbourne Uni	diepstraten.s@wehi.edu.au
2nd author	Savannah	Young	WEHI	young.s@wehi.edu.au
3rd author	John	La Marca	WEHI, Melbourne Uni	lamarca.e@wehi.edu.au
4th author	Zilu	Wang	WEHI, Melbourne Uni	wang.z@wehi.edu.au
5th author	Ruth	Kluck	WEHI, Melbourne Uni	kluck@wehi.edu.au
6th author	Andreas	Strasser	WEHI, Melbourne Uni	strasser@wehi.edu.au
7th author	Gemma	Kelly (CA)	WEHI, Melbourne Uni	gkelly@wehi.edu.au
8th author				
9th author				
10th author				

Please use an additional sheet if there are more than 10 authors.

SPRINGER NATURE Springer Nature is one of the world's leading global research, educational and professional publishers, created in May 2015 through the combination of Nature Publishing Group, Palgrave Macmillan, Macmillan Education and Springer Science+Business Media.

Change of authorship request form - Journals (pre-acceptance)

SPRINGER NATURE

Section 5: Author contribution, Acknowledgement and Disclosures. Please use this section to provide a new disclosure statement and, if appropriate, acknowledge any contributors who have been removed as authors and ensure you state what contribution any new authors made (if applicable per the journal or book (series) policy). Please ensure these are updated in your manuscript - after approval of the change(s) - as our production department will not transfer the information in this form to your manuscript.

New acknowledgements:

NA

New Disclosures (financial and non-financial interests, funding):

Program Grant GNT1113133 to RMK and AS
Leukemia & Lymphoma Society of America (Specialized Center of Research [SCOR] grant no. 7015-18 to RMK, AS and GLK

New Author Contributions statement (if applicable per the journal policy):

RMK provided reagents and critical review of the manuscript.

State 'Not applicable' if there are no new authors.

SPRINGER NATURE Springer Nature is one of the world's leading global research, educational and professional publishers, created in May 2015 through the combination of Nature Publishing Group, Palgrave Macmillan, Macmillan Education and Springer Science+Business Media.

SPRINGER NATURE

Change of authorship request form - Journals (pre-acceptance)

Section 6: Declaration of agreement. *All* authors, unchanged, new and removed *must* sign this declaration.
(NB: Please print the form, (docu)-sign and return/upload a scanned copy. Please note that signatures that have been inserted as an image file are acceptable as long as it is handwritten. Typed names in the signature box are unacceptable.) *** Please delete as appropriate. Delete all of the bold if you were on the original authorship list and are remaining as an author.**

	First name	Family name		Signature	Date
1st author	Sarah	Diepstraten	I agree to the proposed new authorship shown in section 4 /~~**and the addition/removal*of my name to the authorship list**~~ /and the proposed change in corresponding author	[illegible]	16/12/22
2nd author	Savannah	Young	I agree to the proposed new authorship shown in section 4~~**/and the addition/removal*of my name to the authorship list**~~ /and the proposed change in corresponding author	[illegible]	18/12/22
3rd author	John	La Marca	I agree to the proposed new authorship shown in section 4 /**a~~nd the addition/removal*of my name to the authorship list~~** /and the proposed change in corresponding author	[illegible]	18.12.2022
4th authors	Zilu	Wang	I agree to the proposed new authorship shown in section 4 /~~**and the addition/removal*of my name to the authorship**~~**list** /and the proposed change in corresponding author	Zilu Wang	18/12/22
5th author	Ruth	Kluck	I agree to the proposed new authorship shown in section 4 /**and the addition/~~removal~~*of my name to the authorship list** /and the proposed change in corresponding author	[illegible]	18/12/22
6th author	Andreas	Strasser	I agree to the proposed new authorship shown in section 4 /~~**and the addition/removal*of my name to the authorship**~~**list** /and the proposed change in corresponding author	[illegible]	18/12/22
7th author	Gemma	Kelly	I agree to the proposed new authorship shown in section 4 /~~**and the addition/removal*of my name to the authorship list**~~ /and the proposed change in corresponding author	[illegible]	18/12/22

SPRINGER NATURE Springer Nature is one of the world's leading global research, educational and professional publishers, created in May 2015 through the combination of Nature Publishing Group, Palgrave Macmillan, Macmillan Education and Springer Science+Business Media.

	First name	Family name		Signature	Date
8th author			I agree to the proposed new authorship shown in section 4 /**and the addition/removal*of my name to the authorship list** /and the proposed change in corresponding author		
9th author			I agree to the proposed new authorship shown in section 4 /**and the addition/removal*of my name to the authorship list** /and the proposed change in corresponding author		
10th author			I agree to the proposed new authorship shown in section 4 /**and the addition/removal*of my name to the authorship list** /and the proposed change in corresponding author		

Please use an additional sheet if there are more than 10 authors.

In case of author collaborations with formal agreement:

	Name of consortium/consortia	First name	Family name		Signature	Date
Representative/legal guarantor				I agree to the proposed new authorship shown in section 4 /**and the addition/removal*of my name to the authorship list** /and the proposed change in corresponding author		

Both added/removed authors should complete the information in the first table under Section 6.

---- End of form ----

SPRINGER NATURE Springer Nature is one of the world's leading global research, educational and professional publishers, created in May 2015 through the combination of Nature Publishing Group, Palgrave Macmillan, Macmillan Education and Springer Science+Business Media.
