# Supplementary material for: Lymphoma cells lacking pro-apoptotic BAX are highly resistant to BH3-mimetics targeting pro-survival MCL-1 but retain sensitivity to conventional DNA-damaging drugs
Source: Cell Death Differ. 2023 Feb 8;30(4):1005–17. doi: 10.1038/s41418-023-01117-0 (PMC10070326; doi:10.1038/s41418-023-01117-0)
Supplement: Supplementary file 5 — author contribution form [file 41418_2023_1117_MOESM5_ESM.pdf]

DECLARATION OF CONTRIBUTIONS TO ARTICLE

ADMC

Manuscript Number: CDD-22-1867

Journal Name: *Cell Death & Differentiation* (the 'Journal')

Proposed Title of the Contribution:

Lymphoma cells lacking pro-apoptotic BAX are highly resistant to BH3-mimetics targeting pro-survival MCL-1 but retain sensitivity to conventional DNA damaging drugs. (the 'Contribution)

Author(s):

Sarah T Diepstraten, Savannah Young, John E La Marca, Zilu Wang, Ruth M Kluck, Andreas Strasser, Gemma L Kelly (the 'Authors')

For all *CDD* articles, each person named as an author in the published version must be able to show he or she has contributed substantially to the article.

Authorship credit should be based on 1) substantial contributions to conception and design, acquisition of data, or analysis and interpretation of data; 2) drafting the article or revising it critically for important intellectual content; and 3) final approval of the version to be published. Authors should meet conditions 1, 2 and 3.

Any person who cannot be shown to have made a substantial contribution to the article cannot be listed as an author in the final version. The name of any person who is deemed to have made a minor contribution can, however, appear in the Acknowledgments section of the article.

Please complete the table below to indicate the contributions of all named authors to the manuscript.

Author Full Name:	Specification of Contribution to the Manuscript:
Sarah T Diepstraten	Designed and performed experiments, analysed data, drafted manuscript, prepared figures, approved final article.
Savannah Young	Performed experiments, critically edited manuscript, approved final article.
John E La Marca	Performed experiments, critically edited manuscript, approved final article.
Zilu Wang	Generated cell lines used in this study, critically edited manuscript, approved final article.
Ruth M Kluck	Provided reagents and expertise, critically edited manuscript, approved final article.
Andreas Strasser	Designed and supervised project, critically edited manuscript, approved final article.
Gemma L Kelly	Designed and supervised project, critically edited manuscript, approved final article.

Please complete the table below to indicate the contributions of all named authors to the figures.

Figure 1:
Cell lines generated by Zilu Wang, data generated by Sarah T Diepstraten

Figure 2:
Data generated by Sarah T Diepstraten

Figure 3:
Data generated by Sarah T Diepstraten

Figure 4:
Data generated by Sarah T Diepstraten

Figure 5:
Data generated by Sarah T Diepstraten, John E La Marca and Savannah Young

Figure 6:
Data generated by Sarah T Diepstraten, John E La Marca and Savannah Young

Signed for and on behalf of the Author(s):	Print Name:	Date:
G L Kelly	Gemma Kelly	18/12/22
